# Postingestive reward acts through behavioral reinforcement and is conserved in obesity and after bariatric surgery

**DOI:** 10.1371/journal.pbio.3002936

**Published:** 2024-12-17

**Authors:** Gabriela Ribeiro, Ana B. Fernandes, Francisco P. M. Oliveira, João S. Duarte, Manuela Oliveira, Clotilde Limbert, Rui M. Costa, Durval C. Costa, Albino J. Oliveira-Maia

**Affiliations:** 1 Champalimaud Research & Clinical Centre, Champalimaud Foundation, Av. de Brasília, Doca de Pedrouços, Lisboa, Portugal; 2 Lisbon Academic Medical Centre PhD Program, Faculdade de Medicina, Universidade de Lisboa, Avenida Professor Egas Moniz, Lisboa, Portugal; 3 NOVA Medical School, Faculdade de Ciências Médicas, NMS, FCM, Universidade NOVA de Lisboa, Campo Mártires da Pátria 130, Lisboa, Portugal; 4 Department of Endocrinology, Centro Hospitalar de Lisboa Ocidental, Rua da Junqueira, Lisboa, Portugal; 5 Department of Neuroscience and Neurology, Zuckerman Mind Brain Behavior Institute, Columbia University, New York, New York, United States of America; 6 Allen Institute, Seattle, Washington State, United States of America; Max Planck Institute for Metabolism Research, GERMANY

## Abstract

Postingestive nutrient stimulation conditions food preferences through striatal dopamine and may be associated with blunted brain responses in obesity. In a cross-sectional study, we tested flavor-nutrient conditioning (FNC) with maltodextrin-enriched yogurt, with maltodextrin previously optimized for concentration and dextrose equivalents (*n* = 57), and to mask texture cues (*n* = 102). After conditioning, healthy volunteers (*n* = 52) increased preference for maltodextrin-paired (+102 kcal, CS^+^), relative to control (+1.8 kcal, CS^-^) flavors, as assessed according to intake, but not pleasantness. In a clinical study (*n* = 61), behavioral conditioning without effects on pleasantness was confirmed across pre-bariatric candidates with obesity, weight-stable post-surgery patients, and healthy controls, without significant differences between groups. Striatal dopamine D2-like receptor (DD2lR) availability, assessed with [^123^I]IBZM SPECT, was reduced in the obesity group and strongly correlated with conditioning strength and a measure of restrained eating in patients with gastric bypass. These results show that postingestive nutrient stimulation influences human food choices through behavioral reinforcement, and is conserved in obesity and after bariatric surgery.

**Trial Registration:** ISRCTN17965026: Dopaminergic neurotransmission in dietary learning and obesity.

## Abstract 2

Obesity has been associated with altered reward-related eating behavior and brain changes that may be related to overeating. This behavioral and neuroimaging study shows that postingestive nutrient stimulation influences human food choices through behavioral reinforcement and is conserved in obesity and after bariatric surgery.

## Introduction

Postingestive signals about the energy content of food are crucial determinants of food selection, in addition to explicit sensory cues [1–3]. One extensively researched model of how rodents acquire preferences according to postingestive nutrient value is flavor-nutrient conditioning (FNC), with induction of a conditioned preference for an oral flavor, resulting from repeated pairings with the postingestive consequences of a nutrient [1]. FNC protocols have also been tested in humans, with some evidence to support increased liking ratings for nutrient-paired flavors despite considerable methodological challenges [4]. Bland carbohydrates such as maltodextrin [5] have been used to minimize sensory cues and thus isolate postingestive consequences. For example, FNC protocols using flavored beverages, either with added calories from maltodextrin (+112.5 kcal) or non-caloric controls, resulted in modest increases in liking ratings for the former but not the latter [6], and absent effects when either higher or lower doses of maltodextrin were used [7]. However, despite its bland sensory properties, maltodextrin can be detected at different concentrations [5,8], raising concerns about isolating its postingestive effects. Nevertheless, in rodents, there is evidence that the postingestive consequences of sugars can induce ingestive preferences, even in the absence of orosensory input [9,10]. Furthermore, we have shown that the postingestive effects of sucrose sustain food-seeking behaviors that depend on the activity of dopaminergic neurons in the ventral tegmental area (VTA) and are at least partially mediated by the hepatic branch of the vagus nerve [11].

Obesity has been associated with altered reward-related feeding behavior [1,12,13] and brain changes that may be related to overeating, such as lower striatal dopamine D2-like receptors (DD2lR) availability [14] (for review, see Ribeiro and colleagues [15]). Notably, a recent paper suggested that brain responses to postingestive administration of nutrients, including striatal responses and dopamine release, are impaired in patients with obesity and not recovered after moderate, diet-induced weight loss [16]. However, direct comparisons between patients with obesity and lean participants to address the behavioral effects of postingestive nutrients, as well as the impact of bariatric surgery in the former, are lacking. Indeed, bariatric surgery, currently the most effective treatment for severe obesity [17–19], potentially normalizes obesity-related features of reward-related feeding behavior [20–24], with reports of food preference shifts after surgery [20,21,23,25], from energy-dense and palatable foods (e.g., rich in fats and sugars) towards less energy-dense options (e.g., fruits and vegetables). However, the mechanisms underlying these changes are not fully understood [1]. Changes in food reward processing, including postingestive reward, are potentially involved but remain unexplored.

Here, we hypothesized that postingestive reward, as measured in a conditioning paradigm, is impaired in obesity compared to a healthy and lean sample and is recovered by bariatric surgery. To address this hypothesis, we developed a novel FNC protocol in healthy volunteers, fully addressing potential confounders from the orosensory cues provided by maltodextrin. Postingestive conditioning strength obtained in this optimized protocol was then compared between patients with obesity either before or after bariatric surgery and healthy volunteers, all of whom were also assessed with [^123^I]IBZM SPECT to explore potential associations between postingestive conditioning and DD2lR availability. Additional aims included testing the differential impact of bariatric surgery type, i.e., gastric bypass and sleeve gastrectomy, on postingestive reinforcement and DD2lR availability.

## Results

### Study overview

This study was conducted on 272 participants tested in one of 3 main experiments. Conditions for optimal use of maltodextrin in FNC, with identification and masking of the orosensory cues resulting from consumption of maltodextrin solutions, were tested in 159 healthy participants (“Maltodextrin optimization” group), 57 of whom to improve definition of maltodextrin concentration and dextrose equivalents, and 102 to define optimal conditions to mask cues allowing for oral identification of maltodextrin. These initial experiments allowed for the definition of a protocol for FNC, with control for the orosensory identification of maltodextrin. This was applied to 52 other healthy volunteers (the “FNC development” group) to assess behavior in the FNC protocol and perform further optimization. In a final group of 61 participants, to compare patients with obesity either before or after bariatric surgery and a new group of healthy controls, data was collected with the final FNC protocol and nuclear medicine imaging of DD2lR availability. Please see **Table 1** for a detailed demographic and clinical description of participants.

**Table 1:** Demographic, gustatory, and psychometric measures of feeding behavior in healthy subjects.

	Healthy volunteer study			Clinical study				
Variable	Healthy “Maltodextrin optimization” (*n* = 159)	Healthy “FNC Development” (*n* = 52)	*P*-value^1^	Healthy controls (*n* = 27)	Obese (*n* = 11)	Surgical (*n* = 23)		*P*-value^2^
	**Mean (SD) or No. (%)**							
Age, years	36.1 (11.8)	28.5 (7.1)	<0.001	31 (7.7)	41.2 (8.8)	43.8 (11.1)	<0.001	
Gender (male)	55 (34.6%)	15 (28.8%)	0.4	6 (22.2%)	4 (36.4%)	1 (4.3%)	0.06	
BMI, kg/m^2^	23.1 (4)	23.1 (3.2)	0.1	24.7 (2.8)	50.5 (9.5)	29.7 (3.9)	<0.001	
Education, years	16.2 (3)	14.2 (2.6)	0.2	13.8 (2.6)	10.1 (4.3)	9.5 (3.8)	<0.001	
Smokers		12 (23.1%)		4 (14.8%)	2 (18.2%)	2 (8.7%)	0.7	
T2DM	0 (0)	0 (0)		0 (0)	0 (0)	0 (0)		
Hypertension	0 (0)	0 (0)		0 (0)	6 (54.5%)	5 (21.7%)	0.1	
Dyslipidemia	0 (0)	0 (0)		0 (0)	1 (9.1%)	2 (8.7%)	0.97	
Time after surgery (months)						29.7 (3.9)	N.A.	
Taste thresholds, *dB*		4.3 (8.8)		8.3 (9.8)	13.6 (13.2)	15.9 (12.5)	0.1	
Acuity		13.1 (2.5)		14.1 (2)	13.5 (2.1)	13.1 (2.5)	0.3	
Sour ratings, *mm*								
Intensity		57.1 (19.8)		63.9 (15.8)	61.7 (24.2)	60.2 (16.1)	0.8	
Pleasantness		−33.7 (32.6)		−40.6 (24.7)	−35.9 (40.2)	−34.9 (35.5)	0.8	
Salt ratings, *mm*								
Intensity		28.4 (14.6)		37.3 (20.9)	31.1 (20.1)	36.8 (11.8)	0.6	
Pleasantness		−4.6 (18.7)		−12.7 (24.7)	−7.7 (16.7)	−3.9 (18.7)	0.4	
Sweet ratings, *mm*								
Intensity		17.1 (8.0)		20.6 (10.2)	20.9 (8)	18.7 (9.7)	0.8	
Pleasantness		8.97 (9.7)		11.7 (10.82)	15.6 (15.9)	12.9 (11.1)	0.7	
Bitter ratings, mm								
Intensity		40.2 (20.2)		48, (19.2)	42.9 (17.3)	46.4 (17.7)	0.7	
Pleasantness		−36.2 (24)		−42.1 (21.1)	−26.7 (34.2)	−24.2 (33.6)	0.1	
PFS–Aggregate score		2.3 (0.7)		2.3 (0.6)	2.7 (1)	2.1 (0.6)	0.04	
PFS–Food Available		1.9 (0.6)		1.9 (0.6)	2.5 (1.3)	1.7 (0.7)	0.04	
PFS–Food Present		2.9 (1.0)		2.9 (0.9)	2.9 (1.1)	2.6 (1.1)	0.5	
PFS–Food Tasted		2.7 (0.9)		2.7 (0.8)	3.1 (0.7)	2.4 (0.8)	0.1	
YFAS–Diagnosis		0 (%)		0 (0%)	3 (27.3%)	1 (4.5%)	0.01	
YFAS–No. of symptoms		1.5 (1.1)		1.6 (0.9)	3.2 (2)	1.7 (1.3)	0.004	
DEBQ–External Eating		2.8 (0.5)		2.7 (0.4)	2.6 (0.8)	2 (0.6)	0.001	
DEBQ–Restrained Eating		2.4 (0.7)		2.6 (0.8)	2.6 (0.9)	2.8 (0.8)	0.7	
DEBQ–Emotional Eating		2 (0.8)		2.1 (0.6)	2.1 (1.3)	1.6 (1)	0.2	
FARS–Aggregate score		401.2 (43.1)		408.5 (45.3)	358.5 (99.5)	400.3 (39.6)	0.1	

^**1**^Independent samples *T* tests or Pearson’s chi-square (Χ^2^) tests were performed to compare the Healthy “Maltodextrin optimization” and the Healthy “FNC Development” groups.

^**2**^One-way analysis of variance (ANOVA) or Pearson’s chi-square (Χ^2^) tests were performed to compare the healthy controls, obese, and surgical groups.

### Maltodextrin is identified through orosensory cues

**Fig 1:**
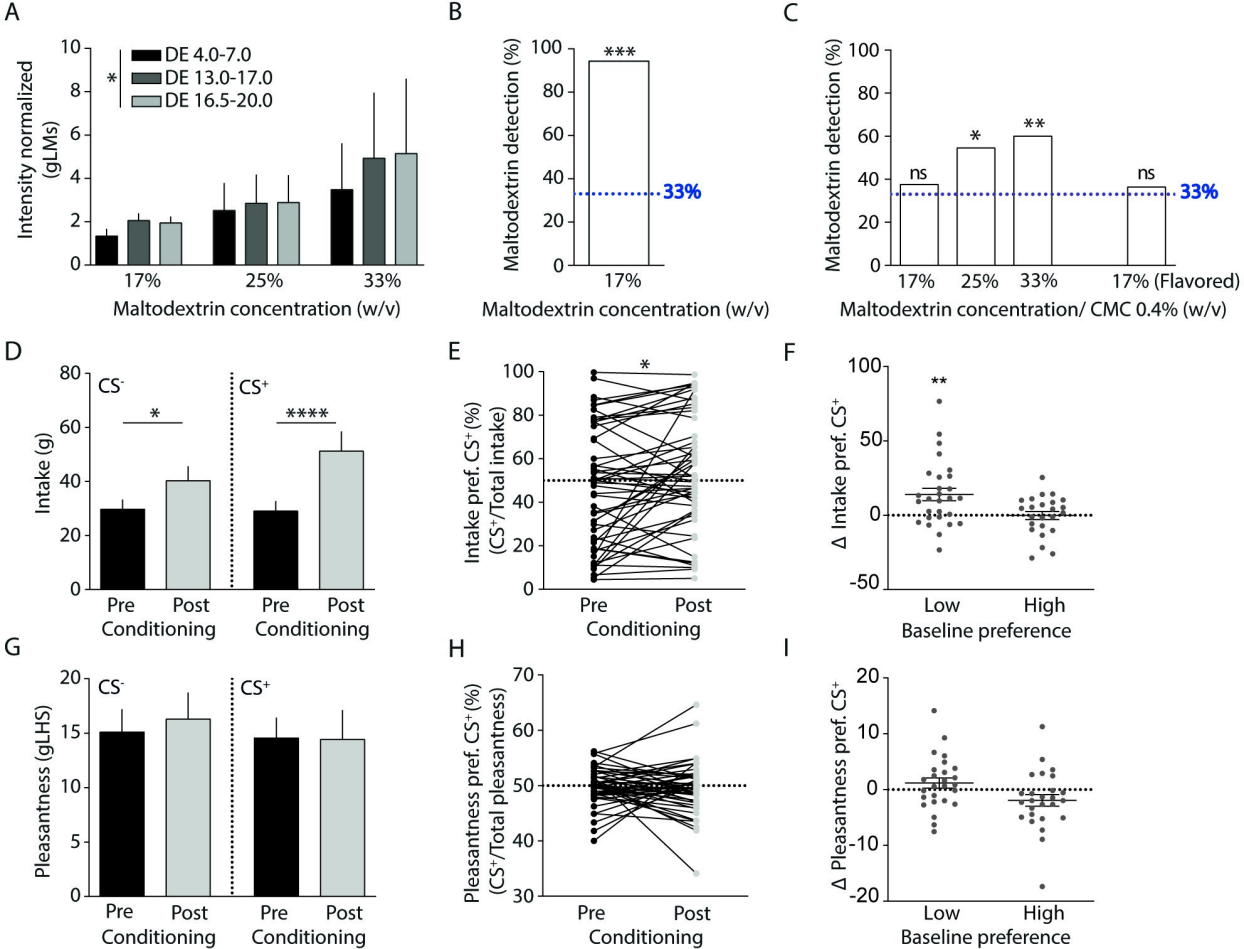
Development of a novel FNC protocol. ** (A)** Intensity and pleasantness ratings of unflavored maltodextrin yogurt were tested across 3 different groups of healthy individuals, each at a different maltodextrin concentration (17%, 25%, or 33% w/v). In each group, participants consumed a base yogurt solution without maltodextrin and 3 solutions with maltodextrin at distinct dextrose equivalents (DE 4–7, DE 13–17, and DE 16.5–20). Intensity ratings of maltodextrin yogurts, normalized to ratings of base yogurt, varied according to DE (F_(2,72)_ = 3.4, *P* = 0.04), independently of the concentration tested (F_(2,36)_ = 0.5, *P* = 0.6; interaction: F_(4,72)_ = 0.7, *P* = 0.6; mixed-model two-way ANOVA; *n* = 39). **(B)** In 3-AFC tests, 1 or 2 of 3 yogurt samples contained maltodextrin (DE4-7), and the other(s) were control yogurts. The percentage of participants that detected maltodextrin was significantly above the chance level (94.4%, *p* < 0.0001; binominal test vs. 33%; *n* = 18). **(C)** In other 3-AFC tests, testing discrimination relative to CMC (0.4% w/v) rather than base yogurt, the higher concentrations of maltodextrin (25% and 33%) were identified significantly above chance (54.5%, *P* = 0.02, *n* = 22; 60%, *P* = 0.001, *n* = 25, respectively), while 17% maltodextrin was not discriminated from CMC (37.5%, *P* = 0.41, *n* = 24), as confirmed when yogurts were flavored (36.4%, *P* = 0.42, *n* = 33). **(D)** After conditioning, while intake of both CS+ and CS- flavors increased significantly (time: F_(1,51)_ = 17.1, *P* = 0.0001; stimulus: *F*_(1,51)_ = 1.2, *P* = 0.3; post hoc tests: CS^+^, *P* < 0.0001, CS^-^_,_ *P* = 0.02), the interaction between factors suggested differential effects between stimuli (F_(1,51)_ = 4.3, *P* = 0.04; repeated measures two-way ANOVA; *n* = 52). **(E)** Post-conditioning, preference for CS^+^, as measured according to intake, increased significantly (t_(51)_ = 2.6, *P* = 0.02, paired *t* test, *n* = 52). **(F)** In participants with low baseline intake preference for CS^+^ (≤50%), there was a significant post-conditioning increase in preference (t_(27)_ = 3.7, *P* = 0.002, *n* = 28), but not in those with high baseline preference (>50%; t_(23)_ = 0.08, *P* = 0.9, *n* = 24, one-sample *t* test vs. 0). **(G)** We found similar pleasantness ratings both for CS^-^ and CS^+^ before and after conditioning, and unchanged preference for CS^+^, as measured by pleasantness **(H)**, irrespective of the baseline preference **(I)**. **Notes:** In panels A, D, F, G, and I, data is presented as mean ± standard error of the mean (SEM). *P ≤ 0.05; **P ≤ 0.01; ***P ≤ 0.001; ****P ≤ 0.0001; ns *P* > 0.05. 3-AFC, 3-alternative forced-choice; CMC, carboxymethylcellulose; DE, dextrose equivalent; FNC, flavor-nutrient conditioning; gLMS/gLHS, general labeled magnitude/hedonic scales. The data supporting this figure is available in S1 Data.

While maltodextrin is typically used in FNC protocols as a source of calories due to insipid taste, we had evidence from preliminary qualitative experiments that sweetness and texture could be cues for detection of maltodextrin solutions. We thus developed a series of experiments to determine conditions in which maltodextrin, when dissolved in low-fat yogurt sweetened with sucralose at 0.01% (w/v), would not be discriminated from the base low-fat yogurt solution without maltodextrin added. Across 39 participants, we started by testing different maltodextrin concentrations and dextrose equivalents (DEs) since there are reports of increasing sweetness for higher DE [26]. Indeed, we found that yogurt intensity ratings, normalized relative to the base yogurt solution without maltodextrin, varied according to maltodextrin DE (4–7, 13–17, and 16.5–20; F_(2,72)_ = 3.4, *P* = 0.04), but not concentration (17%, 25%, and 33% w/v; F_(2,36)_ = 0.5, *P* = 0.6) nor their interaction (F_(4,72)_ = 0.7, *P* = 0.6; *n* = 39; **Fig 1A**). Furthermore, in a separate group of 18 healthy volunteers, even at the lowest maltodextrin DE (4–7) and concentration (17%), in 3-alternative forced-choice (3-AFC) tests with 1 or 2 stimuli consisting of maltodextrin yogurt and the remaining (respectively 2 or 1) of base yogurt, participants very easily identified maltodextrin above chance level (*P* < 0.0001; *n* = 18; **Fig 1B**). Since all yogurts were similarly sweetened with sucralose, and we had evidence from preliminary experiments that texture could be the major cue for maltodextrin detection, we addressed the problem of maltodextrin identification by adding carboxymethylcellulose (CMC; 0.4% w/v), a flavorless and low energy food thickener, to the base yogurt solution. This was tested in additional 3-AFC tests conducted in a new group of 102 healthy volunteers. Discrimination of the CMC-enriched base yogurt was first tested against 17% (*n* = 24), 25% (*n* = 22), and 33% (*n* = 25) of the lowest DE maltodextrin. While 25% and 33%, maltodextrin was still identified significantly above chance level (25.0%, *P* = 0.02, *n* = 22; 33.0%, *P* = 0.001, *n* = 25), the lowest concentration (17%) maltodextrin yogurt was not discriminated from CMC yogurt solutions (*P* = 0.41, *n* = 24). This was conserved when tested in a final group of 33 participants using flavored yogurts, as planned for FNC experiments (*P* = 0.42, *n* = 33; **Fig 1C**). Thus, in subsequent experiments, we used the contrast 17% maltodextrin/0.4% CMC.

### Flavor-nutrient conditioning occurs through increased intake but not pleasantness ratings

In another group of 63 eligible healthy volunteers, we then tested a FNC protocol using the optimized sweetened low-fat yogurt solutions, i.e., with either 17% DE 4.0–7.0 maltodextrin (+0.68 kcal/ml; unconditioned stimulus) or 0.4% CMC (+0.012 kcal/ml; control stimulus), paired to 2 distinct flavors (0.3% w/v, respectively CS^+^ and CS^-^). Conditioning was conducted at home, during 2 days with 150 g CS^+^/Maltodextrin, alternated with 2 days of 150 g CS^-^/CMC. Among these volunteers, 9 were excluded due to errors in the application of or compliance with protocol, and 2 due to low at-home consumption of yogurt, resulting in data from 52 participants for analysis (*n* = 52, **Table 1**). In this group, during at-home conditioning, no effects were found for day or stimulus in this group on hunger, thirst, novelty, intensity, or pleasantness ratings (**S1A–S1E Fig)**. However, there was overall greater intake of CS^+^ than CS^-^ (F_(1,102)_ = 5.5, *P* = 0.02), with less consumption in the second conditioning day (F_(1,102)_ = 8.1, *P* = 0.005; interaction: F_(1,102)_ = 0.5, *P* = 0.5; **S1F Fig**). Hunger, thirst, and intensity ratings also did not differ significantly between pre-and post-conditioning days (**S1G, S1H, and S1J Fig)**, while novelty ratings decreased significantly after conditioning (F_**(**1,51)_ = 10.21, *P* = 0.002), similarly for both stimuli (F_(1, 51)_ = 0.17, *P* = 0.7; interaction: F_(1, 51)_ = 1.14, *P* = 0.29; **S1I Fig)**, presumably as a result of multiple exposures during conditioning. Importantly, intake increased significantly in post-conditioning tests, relative to pre-conditioning (F_(1,51)_ = 17.1, *P* = 0.0001), without significant differences between CS^-^ and CS^+^ (*F*_(1,51) =_ 1.2, *P* = 0.3). However, there was a significant interaction between time and stimulus (F_(1,51)_ = 4.3, *P* = 0.04; **Fig 1D**), showing that this change was differential between stimuli. Indeed, the %preference for CS^+^, as calculated according to intake (intake %preference), significantly increased from pre to post-conditioning (t_(51)_ = 2.61, *P* = 0.02; **Fig 1E**), suggesting that change in this measure (ΔCS^+^ preference = post-test minus pre-test CS^+^ %preference) may be used to assess the efficacy of conditioning per individual. Moreover, the increase in ΔCS^+^ intake preference was particularly evident for participants with low (≤50%) baseline intake %preference for CS^+^ (t_(27)_ = 3.4, *P* = 0.002, *n* = 28), while in those with >50% CS^+^ intake %preference at baseline, ΔCS^+^ preference did not increase nor reduce significantly after conditioning (t_(23)_ = 0.08, *P* = 0.9, *n* = 24; **Fig 1F**). Since the definition of the CS+ yogurt was random, participants were randomly distributed between the high and low preference groups and had similar demographic, gustatory, and psychometric variables **(S1 and S2 Tables**) that are thus not expected to have contributed to differences between the groups. Our results rather suggest that preference could not be further increased by conditioning when it was already high at baseline, with the fact that it also did not decrease arguing against effects resulting simply from regression to the mean [27], and thus supporting that conditioning was restricted to the low baseline preference subgroup. Pleasantness ratings, on the other hand, did not change significantly after conditioning (F_(1, 51)_ = 0.05, *P* = 0.8) and were similar for CS^-^ and CS^+^ (F_(1,51)_ = 0.4, *P* = 0.5; interaction: F_(1,51)_ = 0.2, *P* = 0.8; **Fig 1G**). Consistently, CS^+^ %preference, when calculated according to pleasantness ratings (pleasantness %preference), did not differ between pre- and post-conditioning (*t*_51_ = 0.5, *P* = 0.6; **Fig 1H**). Furthermore, in calculations according to pleasantness measurements, ΔCS^+^ preference did not differ from zero in those with low baseline pleasantness %preference for CS^+^ (t_(25)_ = 1.3, *P* = 0.2, *n* = 26), and had a close to significant reduction, rather than increase, in those with high baseline pleasantness %preference (t_(25)_ = 1.9, *P* = 0.07, *n* = 26; **Fig 1I**). Importantly, a sensitivity analysis excluding participants with BMI 25 kg/m^2^ or greater revealed overlapping results to those obtained in the full sample (**S2A and S2B Fig**). Additionally, the ΔCS^+^ preference measure calculated according to intake was not associated with baseline consumption of milk and yogurt, as self-reported according to average daily number of cups, namely in participants most sensitive to the effects of conditioning (low baseline preference for CS+: r = −0.1, *P* = 0.6, *n* = 28). Overall, these findings support that human flavor nutrient conditioning, when performed controlling for orosensory discrimination of maltodextrin, influences primarily implicit feeding decisions (i.e., increase in intake) rather than explicit assessments of stimuli (i.e., increase of pleasantness).

### Flavor-nutrient conditioning is conserved in obesity and after bariatric surgery

We then used the optimized FNC protocol to test a clinical cohort of patients from a bariatric surgery program, where we recruited 34 eligible patients, 11 of whom with obesity approved for bariatric surgery and 23 after bariatric surgery (gastric bypass, *n* = 13; sleeve gastrectomy, *n* = 10), that were compared with a group of 27 healthy controls (**S3A and S3B Fig**). Groups differed significantly according to age (F_(2,60)_ = 12.7, *P* = 0.00003), BMI (F_(2,60)_ = 106.3, *P* < 0.00001) and years of formal education (F_(2,60)_ = 10.9, *P* = 0.0001; **Table 1**), but significant differences were not found across most gustatory and psychometric variables, except PFS scores (aggregate and food available; both *P =* 0.04), addiction-like feeding behavior (YFAS - number of symptoms, *P* = 0.004; YFAS - diagnosis rate *P* = 0.01), and external eating (*P* = 0.001; **Table 1**).

**Fig 2:**
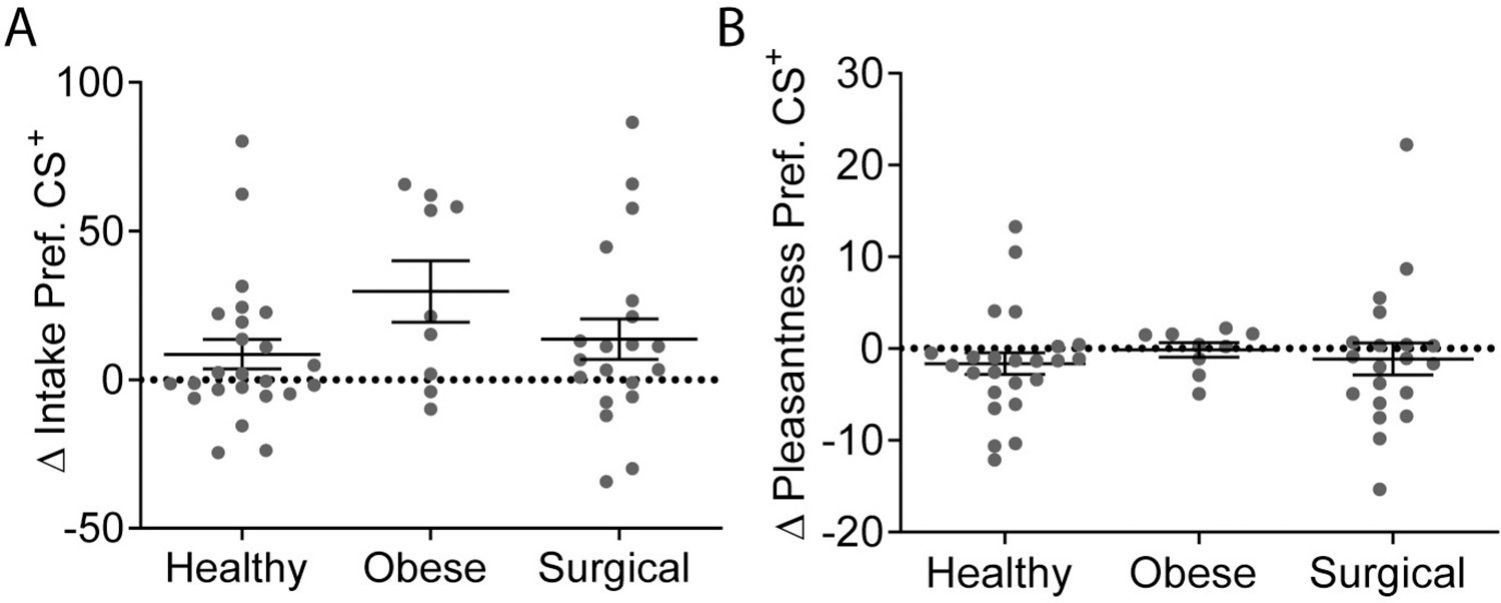
Measures of FNC across the clinical study groups. **(A)** ΔCS^+^ preference, measured by intake, across the clinical study groups. A one-way ANOVA revealed a nonsignificant group effect (F_(2, 50)_ = 1.9, *P* = 0.2) across healthy (*n* = 24), obese (*n* = 9), and surgical (*n* = 20) groups. **(B)** ΔCS^+^ preference, measured by pleasantness ratings, across the clinical study groups. A one-way ANOVA revealed a nonsignificant group effect (F_(2, 50)_ = 0.2, *P* = 0.8) across groups. **Notes:** Data is presented as mean ± SEM. FNC, flavor-nutrient conditioning; gLMS, general labeled magnitude scales; SEM, standard error of the mean. The data supporting this figure is available in S1 Data.

Here, for FNC, flavors with lower baseline intake preference were selected to pair with maltodextrin, given that conditioning was not observed during protocol development when maltodextrin was paired with flavors with high baseline preference (please see **Fig 1F**). Consequently, CMC was paired with the flavor with a higher pre-conditioning intake preference, and maltodextrin was paired with the flavor with a lower preference (**S4 Fig**). During conditioning, significant differences were not found between the groups or CS types regarding hunger, thirst, novelty, and pleasantness ratings (**S5A–S5C and S5E Fig)**. However, intensity ratings varied according to CS type (F_(1, 50)_ = 5.2, *P* = 0.03; **S5D Fig**), while intake varied according to group (F_(1, 50)_ = 7.5, *P* = 0.001; interaction: F_(2, 50)_ = 1.0, *P* = 0.4; **S5F Fig)**. Consistently with data from the original healthy volunteer group, while there were no effects for hunger, thirst, and intensity, novelty ratings decreased from pre- to post-conditioning (*F*_(1,50)_ = 12.9, *P* = 0.001; **S5G–S5J Fig**). Regarding the effects of conditioning on flavor preference, we found that ΔCS^+^ preference, as assessed by intake, increased significantly after conditioning (t_(52)_ = 3.6, *P* < 0.001; *n* = 53), with no significant differences significantly across healthy (*n* = 24), obese (*n* = 9), and surgical groups (*n* = 20; F_(2, 50)_ = 1.9, *P* = 0.2; **Fig 2A**). Regarding pleasantness ratings, ΔCS^+^ preference also did not vary significantly according to group (F_(2, 50)_ = 0.2, *P* = 0.8; **Fig 2B**) and, as observed in the initial experiments, did not reflect any significant effects of conditioning (t_(52)_ = −1.4, *P* = 0.2; *n* = 53). In exploratory analyses, ΔCS^+^ preference did not differ between the sleeve and bypass groups for either intake (t_(18)_ = 1.1, *P* = 0.3) or pleasantness (t_(18)_ = −0.2, *P* = 0.9; *n* = 20). Sensitivity analyses excluding participants with BMI 25 kg/m^2^ or greater from the healthy volunteer group revealed overlapping results to those obtained in the entire sample (**S2C and S2D Fig**). Finally, although there was a significant effect across groups for at-home intake during conditioning, with lower volumes consumed by participants in the surgical group (**S5F Fig**), across all participants as well as healthy, obesity, and surgical groups, we did not find any significant correlation between ΔCS^+^ preference intake and total mean consumption (all: r = 0.2, *p* = 0 .3; healthy: r = 0.2, *p* = 0.3; obese: r = 0.3, *p* = 0.4; surgical: r = 0.1, *p* = 0.6), mean CS+ consumption (all: r = 0.2, *p* = 0.1; healthy: r = 0.2, *p* = 0.3; obese: r = 0.3, *p* = 0.4; surgical: r = 0.3, *p* = 0.2) or mean CS-consumption (all: r = 0.1, *p* = 0.5; healthy: r = 0.2, *p* = 0.5; obese: r = 0.3, *p* = 0.5; surgical: r = −0.1, *p* = 0.8).

### Reduced striatal DD2lR availability in obesity is recovered after bariatric surgery and may be related to postingestive conditioning after gastric bypass

**Fig 3:**
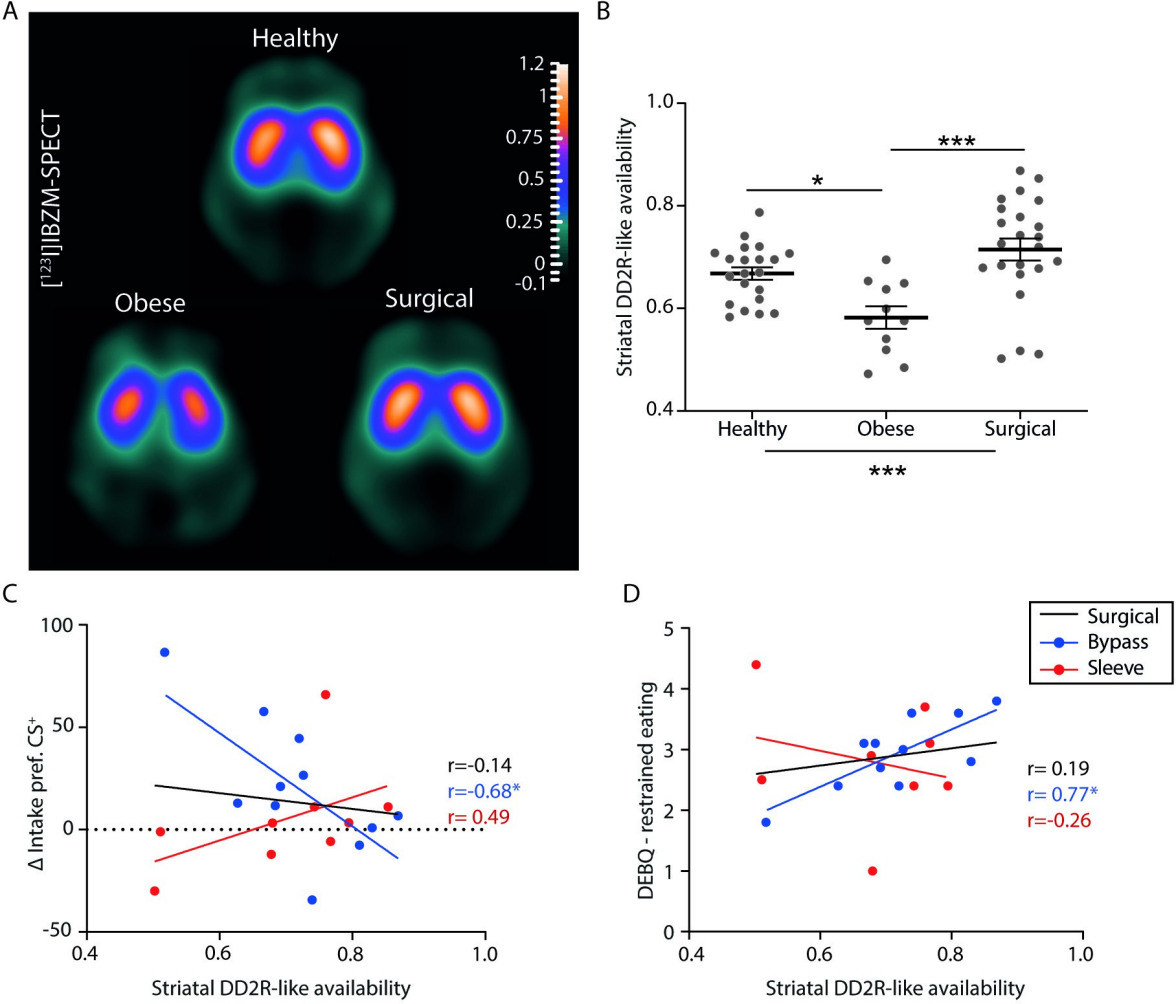
Striatal DD2lR availability in obesity and bariatric surgery and associations with postingestive conditioning and restrained eating. **(A)** Average [^123^I]IBZM group images in the striatal central transverse plane of healthy subjects (*n* = 21; upper panel), patients with obesity (*n* = 11; lower left panel), and surgical patients (*n* = 23; lower right panel). **(B)** There was a group effect for striatal DD2lR availability (F_(2, 52)_ = 9.81, *P* = 0.0002), with post hoc tests supporting lower striatal BP for the obesity group relative to both surgical (*P* = 0.0001) and healthy (*P* = 0.02) groups, but not between surgical and healthy groups (*P* = 0.19). (C) Association between striatal DD2lR availability and ΔCS^+^ preference (intake) across surgery types (Surgical group, r = −0.14, *P* = 0.6, *n* = 20; Bypass, r = −0.68, *P* = 0.02, *n* = 11; Sleeve, r = 0.49, *P* = 0.18, *n* = 9). (D) Association between striatal DD2lR availability and the Dutch Eating Behaviour Questionnaire—restrained eating scores across surgery types (Surgical group, r = 0.19, *P* = 0.4, *n* = 19; Bypass, r = 0.77, *P* = 0.01, *n* = 11; Sleeve, r = −0.26, *P* = 0.5, *n* = 8). Notes: In panel B, data is presented as the mean ± SEM. *P ≤ 0.05; ***P ≤ 0.001. The data supporting this figure is available in S1 Data. DD2lR, dopamine D2-like receptor; SEM, standard error of the mean.

While reduced availability of DD2lR is associated with extreme obesity [14,28–30], there is controversy relative to the possibility that bariatric surgery may normalize this effect [31–34], with a need for further studies and some evidence of advantages in the use of [^123^I]IBZM SPECT [15]. We used this method to assess DD2lR availability in the groups of the clinical study and performed exploratory analyses to test associations with FNC in the surgical group. We found significant differences in DD2lR availability across study groups (F_(2,52)_ = 9.8, *P* = 0.0002), which was lower in patients with obesity (*n* = 11) when compared both with healthy volunteers (*P* = 0.02, *n* = 21) and surgical patients (*P* = 0.0001, *n* = 23), but did not differ between the surgical and healthy groups (*P* = 0.2; **S3A and S3B Fig**), with globally overlapping results in sensitivity analyses excluding participants with BMI 25 kg/m^2^ or greater from the healthy volunteer group (**S2E Fig**). While we did not find differences in DD2lR availability between the gastric bypass (*n* = 13) and sleeve gastrectomy (*n* = 10) subgroups (*P* = 1.0), within the former group DD2lR availability was negatively correlated with ΔCS^+^ preference (intake) (r = −0.7, *P* = 0.02, *n* = 11; **Fig 3C**), and positively correlated with DEBQ—restrained eating (r = 0.8, *P* = 0.01, *n* = 11; **Fig 3D**). Neither of these correlations was found in sleeve gastrectomy (**Fig 3C and 3D**) or other groups (see **S3 Table** for details). Our results corroborate previous findings of decreased DD2lR availability in obesity and support the possibility that these obesity-related effects are reverted by bariatric surgery, similarly following gastric bypass and sleeve gastrectomy. Nevertheless, and in support of specificities of gastric bypass effects on postingestive conditioning, only after gastric bypass was there evidence of associations of DD2lR availability with FNC conditioning strength, as well as with restrained eating.

## Discussion

We showed that flavor-nutrient conditioning, when performed while controlling for explicit sensory effects of maltodextrin, is expressed primarily through implicit consumption decisions rather than explicit assessments of flavor pleasantness ratings. Furthermore, this measure of postingestive learning was conserved across healthy volunteers, patients with severe obesity, and patients treated with bariatric surgery. Measures of DD2lR availability collected in the same clinical cohort were confirmed to be lower in patients with obesity than in healthy volunteers and patients after bariatric surgery, suggesting that obesity-related effects on DD2lR availability are reversible. Importantly, exploratory analyses showing, in the gastric bypass group only, associations between DD2lR availability and conditioning strength, as well as a measure of feeding behavior regulation, suggest that processes of postingestive reinforcement may be of mechanistic relevance for this surgery type.

A central finding of our work is that the acquisition of preferences for flavors paired with calories from maltodextrin occurred according to food intake but not pleasantness ratings. Earlier results of FNC protocols with a similar conditioning period (4 days) and oral maltodextrin as a caloric source (112.5 kcal) reported a small but significant increase in hedonic ratings for CS^+^ flavors [6,7]. Those findings are consistent with a process whereby postingestive stimuli generate a hedonic value (i.e., “liking” or induced sensation of pleasure) [2,35], interacting with other explicit components of food intake, such as flavor perception [2]. Those studies also used triangulation tests to address explicit orosensory detection of maltodextrin. However, they used these tests at the individual participant level to exclude those that could detect maltodextrin, while we analyzed triangulation tests across many individuals to minimize detection of maltodextrin in the study population. Determining and optimizing the conditions under which maltodextrin would not be detected across individuals was a fundamental step of our study, compared to previous studies, and ensured that the conditioning results were determined primarily by postingestive stimulation. Indeed, when conducted under these conditions, FNC resulted in changes in intake of the flavor conditioned with maltodextrin, but not changes in hedonic ratings of pleasantness. Consistently, preclinical research supports the mediation of postingestive signals by striatal dopamine release [9,11,36], which is thought to modulate food-seeking behaviors [11] (i.e., “wanting” or increased effort to obtain a reinforcer), likely at an implicit level [2,35], as is supported here.

We confirmed the results of preference change for CS^+^ flavors according to intake, as well as absent effects on pleasantness ratings, in a distinct cohort of patients from a bariatric surgery clinic and healthy controls. In addition, we found that postingestive conditioning was conserved across the several groups, without evidence for significant variation of the conditioning strength. Since a chronic, excessive caloric intake marks obesity [1,17], it was plausible to hypothesize that patients with severe obesity would show altered conditioning strength relative to controls. Indeed, others have recently published data to support that brain responses to postingestive nutrient stimulation are attenuated or absent in patients with obesity and suggested that these impairments may contribute to overeating [16]. Given the reconfiguration of the gastrointestinal tract induced by bariatric surgery [37] and the resulting self-reported changes in food preferences [20–23], we had hypothesized that bariatric surgery would also impact postingestive reinforcement. Our results do not provide robust support for either of these hypotheses. We did not find deficits in conditioning strength associated with morbid obesity nor changes resulting from bariatric surgery. Indeed, a qualitative inspection of our results suggests that there may be enhanced, rather than impaired, postingestive conditioning in obesity, which is consistent with data in animal models, showing enhanced FNC in rats, as measured in two-bottle tests in rats with diet-induced weight gain, relative both to rats that did not gain weight, and controls maintained on regular chow [38]. The two-bottle test paradigm used in rodents entails relatively low effort and is quite similar to the conditioning measurement used here. Interestingly, other studies have shown that rats exposed to a high-sucrose diet are less motivated to lever press for sucrose rewards in a progressive ratio task [39], and there is also evidence to support deficits in effort-based performance [40], as well as in associative learning tested in more challenging behavioral tasks [41], in patients with obesity. Further work will be necessary to assess the impact of obesity on postingestive conditioning in more demanding behavioral paradigms, for example, implying greater effort to access food reinforcers. Additionally, our clinical study was small, and there was substantial variability in preference data, which may have limited power to identify differences between groups. A larger sample of individuals with obesity and optimized procedures to study postingestive conditioning may be needed for further research on these questions.

Of note, although changes in food preferences have been suggested as a potential mechanism underlying bariatric-induced weight loss, and results from self-reported data suggest a shift away from high-sugar/fat foods to options with lower calorie density and palatability, a direct assessment using an ad libitum paradigm under residential conditions failed to replicate post-bariatric shifts in food preferences [42]. That work showed that the decrease in energy intake was driven primarily by a reduction in portion size. Furthermore, there were reductions in explicit liking and implicit wanting for sweet foods that were not associated with reductions in the intake of sweet foods [42]. Others have also shown that the decrease in meal size after gastric bypass surgery was mainly due to a reduction in average eating burst size and overall meal duration [43]. Our results of unaltered postingestive conditioning following bariatric surgery are thus in line with results from others directly assessing ingestive behavior and supporting altered meal size, rather than modified food preferences, following bariatric surgery [42,43].

Previous findings of lower DD2lR availability in patients with obesity when compared with healthy individuals, described with similar methods as those used here [44] or with [^11^C]raclopride positron emission tomography (PET) [45], were confirmed, as well as the association of BMI and DD2lR availability among patients with morbid obesity [15,45]. Our findings of higher DD2lR availability in the surgical group are globally consistent with a previous study using [^123^I]IBZM SPECT and describing a significant increase in DD2lR availability in women with morbid obesity 2 years after gastric bypass, but still with reduced levels in comparisons with age-matched controls [33]. We found a complete reversal to levels similar to those in healthy volunteers on average 2.5 years after surgery, which may have been due to study design and/or to greater diversity in our sample. Indeed, we did not restrict recruitment only to women, and studied patients treated with gastric bypass as well as sleeve gastrectomy groups. While DD2lR availability did not differ between surgery types, this may have contributed to small differences relative to published data. DD2lR availability, as assessed here with [^123^I]IBZM SPECT, is a static representation of dopaminergic physiology, and the changes associated with obesity may thus reflect decreased expression of the receptor and/or greater occupancy by dopamine [14]. van Galen and colleagues [16] described impairments of the striatal dopamine response to intragastric lipids, but not glucose, in patients with obesity, which, despite the absence of direct comparisons with healthy volunteers, is not suggestive of enhanced dopamine release [16]. In animal research, down-regulation of dopamine D_2_ receptors was shown to result from consumption of energy-dense diets [46,47], but data on the impact of obesity on dopamine responses to food is lacking. Additional research is needed to fully understand the association between obesity, weight loss, and dopamine homeostasis.

Importantly, in exploratory analyses, we found a moderate to strong inverse association between DD2lR availability and postingestive conditioning strength in the gastric bypass group only. In the same surgical group, DD2lR availability had a strong direct association with restrained eating that, in turn, had a moderate to strong negative correlation with conditioning strength. Volkow and colleagues [48] showed that high-restrained eaters had more significant striatal dopamine responses to food stimulation, as assessed with [^11^C]raclopride PET, with higher restraint suggested to reflect a preventive adaptation strategy to minimize exposure to salient food cues [49]. While, to our knowledge, there are no studies addressing the effects of gastric bypass on striatal dopamine responses, our results suggest that, after gastric bypass, patients with the largest increase of DD2lR availability are also those with greater use of restraint as a coping behavior and with the least sensitivity to postingestive conditioning. These associations were absent for sleeve gastrectomy, where nonsignificant correlations in the opposite direction were found. Other studies have described the differential effects of gastric bypass and sleeve gastrectomy on food reward-related measures [21]. It is possible that distinct methods for bariatric surgery, as well as variations within the same surgery type, may alter the vagal mediation of postingestive signals [11]. However, evidence to support the importance of the vagus nerve for weight loss and appetite suppression after gastric bypass, collected in rodents, is mixed [50,51]. Further research, specifically designed to test the importance of postingestive reinforcement in the context of gastric bypass, is needed to address the hypotheses raised here.

### Limitations

The results of this study should be interpreted according to its limitations. Our FNC protocol is limited by the fact that the 4 conditioning days were conducted at home to avoid loss of follow-up, but it also limits experimental control over this phase of the experiment. Despite our efforts to minimize these limitations, namely the use of saliva samples to increase compliance for fasting and exclusion of participants with low adherence to conditioning procedures, there is a possibility for self-report bias that we cannot account for. On the other hand, we did not perform a priori group matching in the clinical study for age, gender, or education. A strictly matched case-control design in the clinical study would have been an asset but is hindered due to the challenges in recruitment within the clinical groups, particularly for a complex protocol as described here. Furthermore, the obesity and surgical groups were not assessed prospectively to avoid the effects of learning on repeated exposures to the FNC paradigm and due to the known challenges of longitudinal follow-up in this clinical population [52]. Challenges in recruitment of the clinical population were also reflected in small sample sizes that may have limited statistical power. Larger studies addressing more restricted hypotheses (e.g., differences in obesity versus controls or the impact of gastric bypass) are needed to replicate and expand these results.

It is also noteworthy that we applied exclusion criteria directly related to metabolic health, namely diabetes, and treatment with antidiabetic medication, contributing to a sample not fully representative of bariatric populations. Indeed, we have previously described that approximately 20% of pre-bariatric patients recruited from the Portuguese healthcare system have T2DM [24]. This decision resulted from concerns that experimental instructions for fasting would not be safe in patients with T2DM. However, glycemic metabolism may likely impact FNC processes, and future studies will benefit from including a more extensive metabolic characterization, including measures of glucose homeostasis, among others, in addition to direct measures of ingestive behavior and other variables that allow phenotyping of the patients. Finally, we assessed DD2lR availability in a static protocol, after the FNC protocol. Ideally, future research should quantify brain responses to postingestive conditioning in real time.

## Conclusions

Using a novel method for FNC in humans, we showed that postingestive reinforcement occurs in healthy subjects and is expressed in implicit behavior rather than explicit pleasantness scores. Furthermore, this postingestive learning was conserved in patients with obesity and post-bariatric patients, suggesting that it may play a role in feeding behavior regulation across these groups. However, reduced DD2lR availability was found in patients with obesity when compared to post-bariatric patients, as well as healthy volunteers, with associations between this variable and postingestive conditioning strength, specifically for patients treated with gastric bypass. Thus, postingestive nutrient stimulation acts through implicit behavioral reinforcement rather than explicit modulation of food pleasantness, is conserved in obesity and after bariatric surgery, and may play a role in the impact of gastric bypass on feeding behavior regulation.

## Methods

### Study design and participants

Healthy volunteers were recruited from the community to optimize the conditions for using maltodextrin in FNC (“Maltodextrin optimization” group) and then to test and optimize a controlled protocol for FNC (“FNC development” group). Inclusion criteria were age between 18 and 65 years and general good health as determined by the investigator. Exclusion criteria assessed at entry into the study were active acute respiratory infection, active neurological or psychiatric disease; active gastrointestinal, hepatic, or pancreatic disease; diabetes, illicit substance use or alcohol abuse; use of any neuropsychiatric medication (including anxiolytics, antipsychotics, antidepressants, anticonvulsants, stimulants, anti-dementia medication, dopamine agonists, and opioid analgesics) or antidiabetic medication (including glp-1 agonists); illiteracy, or otherwise not understanding instructions for the study; prior major gastrointestinal surgery and intra-gastric balloon in the previous 12 months; history of food allergies, including any allergy to milk components or lactose intolerance; pregnancy or breastfeeding. The clinical cohort consisted of consecutive patients at a tertiary care outpatient center specialized in the surgical treatment of obesity, belonging to Centro Hospitalar de Lisboa Ocidental, E.P.E., in Lisbon, Portugal. The cohort included patients approved for bariatric surgery and on the waiting list (obese group) and patients who had received bariatric surgery (surgical group). The latter were recruited no less than 1.5 and no more than 4 years after either gastric bypass or sleeve gastrectomy, when patients are expected to be weight stable and capable of consuming small volumes of liquid. Approval for bariatric surgery followed standard criteria defined by the Portuguese National Health Service [24]. Exclusion criteria for patients were equivalent to those mentioned above, except for BMI and prior major gastrointestinal surgery for the surgical group only. Patients were identified by the clinical team, and those consenting to be contacted were screened by phone. Those not excluded were further assessed for eligibility at admission into the study. For patients, we retrieved the surgery date and type from clinical files to avoid self-report bias. An additional group of healthy volunteers was recruited for comparison with patients. Exclusion criteria were equivalent to those mentioned above, as well as obesity (BMI ≥ 30 kg/m^2^) and underweight (BMI < 18.5 kg/m^2^). Participants of the study were recruited between 11/01/2013 and 06/12/2021, with the recruitment for the clinical study starting on 30/12/2016. The study was conducted according to the principles expressed in the Declaration of Helsinki. Approval for the study protocol was granted by Ethics Committees of the Champalimaud Foundation (ref: none available), the Lisbon Academic Medical Centre (ref: 124/16), and Centro Hospitalar Lisboa Ocidental (ref: none available). Written informed consent was obtained from each participant. The possibility of discontinuing participation at any time during the study was given to all participants. All data were de-identified.

### Solutions

Yogurt (34 kcal, 0.1 g of fat, 4.3 g of carbohydrates, and 4.0g of protein per 100 g) was purchased from a national commercial provider (Continente, Portugal) and was stored at 4°C. Sucralose, maltodextrin, carboxymethylcellulose (Sigma Aldrich), and flavors (Nature’s Flavors, Orange, California, United States of America) were stored at room temperature. Milli-Q water was obtained from our institutional distilled water system. All yogurt-based solutions were prepared daily under sterile conditions and stored at 4°C until 1 h before each experiment when they were transferred to room temperature. Maltodextrin was first diluted in 1/3 of the intended final yogurt solution volume in Milli-Q water. The solution was dissolved using a plate heater and a magnetic stirrer at 90°C (Ohaus, USA). Once this solution was again at room temperature, the final intended volume was completed with 2/3 yogurt to obtain final maltodextrin yogurt at concentrations of 17%, 25%, or 33% (w/v; respectively 0.68 kcal/ml, 1 kcal/ml, 1.32 kcal/ml). CMC (carboxymethylcellulose) yogurt solutions were similarly prepared to obtain a final concentration of 0.4% (w/v; (0.012 kcal/ml). A base yogurt solution was prepared with 1/3 Milli-Q water and 2/3 yogurt (v/v). All yogurt solutions had sucralose added at a concentration of 0.01% (w/v). For flavored solutions (cashew, lychee, tamarind, cider, black currant, and pomegranate), the selected flavor was added at a concentration of 0.3% (w/v).

### Optimizing maltodextrin concentration and dextrose equivalents

In the first cohort of healthy volunteers, psychophysical assessments of distinct maltodextrin concentrations and dextrose equivalents were performed. Participants were divided into 3 groups, each testing one concentration of maltodextrin (17%, 25%, or 33%). For each concentration, in addition to a base yogurt solution, participants sampled 3 solutions of maltodextrin-enriched yogurt, all at the same concentration but prepared with maltodextrin of distinct DEs, namely DE 4–7, DE 13–17, and DE 16.5–20. The 4 yogurt solutions were presented in randomized order and immediately rated according to intensity (0 to 100 mm general Labelled Magnitude Scale—gLMS [53]) and pleasantness (−100 to 100 mm, general Labelled Hedonic Scale—gLHS [54]).

### Discrimination tests to assess maltodextrin detection

Additional groups of healthy volunteers performed a 3-AFC test to determine the discriminability of sweetened maltodextrin yogurt solutions. In an initial test, discrimination of 17% DE 4–7 maltodextrin was tested against a base yogurt solution. The 3-AFC test presented 1 or 2 cups with 17% maltodextrin yogurt solution, with the remaining (respectively 2 or 1) cup(s) containing the base yogurt solution. Participants were asked to sample all 3 of the yogurts and then select the one that was different from the other two according to any sensory attribute that was salient for that participant (e.g., taste, smell, texture, among others). In 3 additional cohorts of healthy volunteers, each of the 3 different maltodextrin yogurt concentrations (17%, 25%, or 33%), all prepared with DE 4–7 maltodextrin, were contrasted in 3-AFC tests with CMC yogurt solution (0.4%). In a final group of healthy participants, the contrast between 17% maltodextrin and 0.4% CMC yogurt solutions was repeated, but with one of 6 flavors (cashew, lychee, tamarind, cider, black currant, and pomegranate; 0.3%) added to the solutions used in each 3-AFC test.

### Flavor-nutrient conditioning

Experimental sessions occurred on 6 consecutive days following an overnight fast of 8 to 10 h. Participants attended the laboratory on the first (pre-conditioning) and last (post-conditioning) days, with 4 conditioning days performed at home between the first and last test days. During the experiments, Milli-Q water at room temperature was available for consumption if desired by the participant. On the first day, participants were assessed for height and weight with a digital scale and a mechanical stadiometer (Kern & Sohn GmbH, Balingen, Germany) with light clothes and without shoes. At the end of that day, each participant completed gustatory psychophysics (taste strips method for citric acid, sodium chloride, sucrose, and quinine hydrochloride; taste thresholds assessed with electrogustometry [55]) and psychometric evaluation of reward-related feeding behavior (Power of Food Scale [13,49,56,57], Yale Food Addiction Scale [58,59], Dutch Eating Behavior Questionnaire [60,61], and Food Action Rating Scale [62]), as described previously (please see Ribeiro and colleagues [24] for details). On the pre-conditioning day, after collecting ratings of hunger and thirst on 0 to 100 mm Visual Analogue Scales (VAS), participants were presented with samples of 6 differently flavored (cashew, lychee, tamarind, cider, black currant, and pomegranate) CMC yogurt solutions, presented in random order for ratings of stimulus novelty (0 to 100 mm VAS, with higher numbers indicating greater novelty), intensity (0 to 100 mm gLMS, with higher numbers indicating greater intensity), and pleasantness (−100 to 100 mm gLHS, with more positive numbers indicating greater pleasantness, and more negative numbers indicating greater unpleasantness). We selected 2 flavored beverages for each participant based on high novelty and similar moderate pleasantness. Each subject then performed 6 trials of 3-AFC discrimination tests contrasting the 2 selected flavors, with a revision of the flavors selected if correct discrimination was not obtained in at least 4 trials. Participants were excluded if 2 flavors with pleasantness rated above 0 in the gLHS, and that were correctly discriminated in 3-AFC tests, could not be identified. We then presented the 2 flavored CMC yogurt solution in 2 large white cups for ad libitum consumption/intake and measured weight (g) before and after consumption (intake measurement). In the initial cohort of healthy volunteers, one of these flavors was randomly chosen to pair with maltodextrin during home conditioning (CS^+^, +102 kcal), while the alternate flavor was paired with CMC (CS^-^, +1.8 kcal). For the clinical experiment, maltodextrin was always paired with the least preferred flavor, as assessed according to intake during ad libitum consumption. In the following 4 conditioning days, at home, subjects were instructed to maintain overnight fasting, after which they should consume yogurt solutions, and refrain from eating anything else for the following hour. Yogurts for at-home consumption were distributed to participants in the pre-conditioning day in sterilized glass bottles, containing 150 g of yogurt, that they were instructed to conserve at 4°C. Bottles were labeled with the consumption date and a letter code assigned to CS^+^ or CS^-^ flavors, so that they consumed either a 17% maltodextrin yogurt solution, paired with the CS^+^ flavor, in 2 non-consecutive days, or a 0.4% CMC yogurt solution, paired with the alternate flavor (CS^-^), in the 2 alternate days, with the order of flavors randomized. Participants were instructed to consume the maximum yogurt possible and return any yogurt that they did not consume for quantification of intake (g). Pre-conditioning, conditioning, and post-conditioning data were excluded from analysis in participants for whom mean home consumption was, on average, less than 25 g in total or for any of the 2 flavors. They were also asked to perform ratings of hunger and thirst prior to consumption, and of stimulus novelty, intensity, and pleasantness after consumption. In the post-conditioning day, we presented the same six-flavor sequence of CMC yogurt solutions, as on the first day, for the same process of flavor rating. Then, the 2 flavors selected for conditioning were given for ad libitum consumption. The main outcomes of this protocol were changes in %preference for CS^+^, assessed according to intake (CS^+^ intake %preference) or pleasantness ratings (CS^+^ pleasantness %preference) from pre- to post-conditioning. In participants of the clinical study, at the end of the last day, single-photon emission computed tomography (SPECT) scans were performed.

### Striatal DD2lR availability imaging

We assessed striatal DD2lR availability using SPECT with [^123^I]-Iodobenzamide ([^123^I]IBZM, GE Healthcare, Eindhoven, NL). Participants were scanned early in the afternoon for approximately 30 min, 2 h after a bolus injection of 185 MBq of [^123^I]IBZM. Each participant was pretreated with potassium iodide to block thyroid uptake of free radioactive iodine (^123^I). SPECT imaging was performed using a Philips BrightView gamma camera (Philips Healthcare, Eindhoven, NL) with low-energy and high-resolution collimators. Image reconstruction was performed using the Astonish algorithm (Philips Healthcare, Eindhoven, NL), an optimized 3D ordered subsets expectation maximization (3D-OSEM) algorithm. After reconstruction, images were corrected for attenuation using the Chang method (linear attenuation coefficient of 0.11 cm^-1^) and the Hanning filter (cut-off 1.0). SPECT images were reconstructed with cubic voxels of 4.664 mm width, and a region of interest (ROI) analysis was performed for quantification based on an automated software validated for brain [^123^I]FP-CIT SPECT scans [63]. This software was adapted for brain [^123^I]IBZM and validated against semiautomated quantification performed by experienced nuclear medicine physicians. The primary outcome of ROI analysis was the striatal binding potential (BP) in the ROIs, which is a proxy for striatal DD2lR (i.e., D2 and D3 receptors) binding. When the radiopharmaceutical reaches equilibrium, the BP can be obtained as the ratio of the specific uptake in the target region and the nonspecific uptake, as shown in **Eq 1**.


$$BP=\frac{mean\;counts\;per\;voxel\;in\;the\;target\;region-mean\;counts\;per\;voxel\;in\;the\;reference\;region}{mean\;counts\;per\;voxel\;in\;the\;reference\;region}$$
**Eq 1.** Binding potential.

The reference region was a portion of the occipital lobe where D2 and D3 receptors are absent. The software quantifies the BP in 6 striatal ROIs (right caudate, right putamen, right striatum, left caudate, left putamen, and left striatum). For statistical analyses, the left and right striatum mean values were considered.

### Data analysis

Analyses were performed using SPSS version 29 (SPSS Inc., Chicago, Illinois, USA). Graphs were produced in GraphPad Prism version 8.0 (GraphPad Software, La Jolla, California, USA) and edited in Adobe Illustrator version 2022 (Adobe Inc., San Jose, Califonia, USA). Data for continuous measurements is presented as mean ± standard error of the mean (SEM). Assessment of the normal distribution of continuous measurements was performed according to visual inspection of distribution as well as analysis of kurtosis, skewness, and comparison between mean and median. To address items with missing values, for scales where this approach is admissible (PFS, DEBQ, and FARS), simple imputation was performed with the mean score of the respective subscale when missing values represented 10% or less of the total items in that subscale. Demographic, clinical, psychometric, and psychophysical data was compared between groups using independent samples *t* tests or one-way analysis of variance (ANOVA) for continuous variables and χ2 tests for categorical variables. In the maltodextrin optimization group, normalized intensity ratings were analyzed using mixed model two-way ANOVA according to concentration (between subjects) and maltodextrin DE (within subjects). The proportion of participants correctly discriminating maltodextrin yoghurts in 3-AFC tests was contrasted to 1/3 (chance level) using binomial tests. To determine changes in raw intake and pleasantness ratings from pre- to post-conditioning in the healthy group (“FNC development”), we used repeated-measures two-way ANOVA with intake (g) or pleasantness ratings (mm) as independent variables, according to a time factor pairing pre- to post-conditioning days (pre-post) and a stimulus factor comprising CS^-^ or CS^+^ flavors (CS^*-*^ versus CS^*+*^). Intake %preference for CS^+^ was calculated as [CS^+^ intake/(CS^-^ + CS^+^ intake) *100]. The same formula, but using gLHS pleasantness ratings, was used to calculate pleasantness %preference for CS^+^. In this case, we transformed pleasantness ratings by adding the amount needed for the minimum value to be 1 (“+101”). To determine changes in preference for CS^+^, we used paired *t* tests to compare pre- to post-preferences according to intake or pleasantness. The difference between the pre-and post-conditioning preference for CS^+^ (Δpreference CS^+^), calculated according to either intake or pleasantness, was analyzed separately according to respective baseline %preference, namely low (<50%) and high (≥50%), using one-sample *t* tests contrasting against zero, to test whether there were significant changes in preference in each group. To compare groups in the clinical cohort for Δpreference CS^+^ (intake) or DD2lR availability, we used a one-way ANOVA. Other data from FNC experiments either comparing pre- versus post-conditioning days (hunger, thirst, novelty, intensity) or data from home-conditioning (hunger, thirst, novelty, intensity, pleasantness, intake) was analyzed using repeated-measures two-way ANOVA, paired *t* tests or mixed-model two-way ANOVA in the case of between-group analyses. Across ANOVA analyses, Bonferroni post hoc tests were performed as planned. Exploratory associations between DD2lR BP, Δ intake preference for CS^+^, and gustatory and psychometric feeding behavior variables were determined using Pearson’s correlation (r). A two-tailed *p*-value of 0.05 was selected as the significance level for all analyses. Further details on the primary statistical models performed are described in **S4 Table**. As shown in **S5 Table**, we determined Eta-squared (η [2]) to measure effect size in the analysis of variance (ANOVA) and mean differences with 95% CIs and Cohen’s d to estimate the effect size of the mean’s differences (Cohen’s d = (M_2_—M_1_) / SD_pooled_) [64]. Regarding correlations, the r^2^ was calculated as the measure of the effect size of correlations. The magnitude of effect size according to Cohen’s (1988) classification, namely, small (d ≤ 0.2), medium (0.2 < d < 0.8), and large (d ≥ 0.8) [64], is shown in S5 Table.

## Supporting information

S1 STROBE Checklist STROBE Statement—Checklist of items that should be included in reports of cross-sectional studies. (DOC)

S1 Fig Complementary conditioning measures in healthy subjects. Across conditioning for CS^-^ and CS^+^ flavors, ratings did not vary according to stimulus or conditioning day for (**A**) **Hunger** (Stimulus: F_(1,102)_ = 0.04, *P* = 0.8; Day: F_(1, 102)_ = 3.0, *P* = 0.08; Interaction: F_(1,102)_ = 9.9, *P* = 0.9), **(B) Thirst** (Stimulus: F_(1,102)_ = 0.1, *p* = 0.8; Day: F_(1, 102)_ = 3.2, *P* = 0.07; Interaction: F_(1,102)_ = 0.08, *P* = 0.8), (**C**) **Novelty** (Stimulus: F_(1,102)_ = 0.002, *P* = 0.9; Day: F_(1, 102)_ = 2.7, *P* = 0.1; Interaction: F_(1,102)_ = 1.3, *P* = 0.3), (**D**) **Intensity** (Stimulus: F_(1,99)_ = 0.3, *P* = 0.6; Day: F_(1, 99)_ = 0.09, *P* = 0.8; Interaction: F_(1,99)_ = 1.2, *P* = 0.3), and (**E**) **Pleasantness** (Stimulus: F_(1,98)_ = 0.9, *P* = 0.3; Day: F_(1,98))_ = 0.4, *P* = 0.5; Interaction: F_(1,98)_ = 1.9, *P* = 0.2). **(F) Intake** volumes were higher for CS^+^ than CS^-^ (F_(1,102)_ = 5.5, *P* = 0.02), and decreased across conditioning days (F_(1,102)_ = 8.1, *P* = 0.005; Interaction: F_(1,102)_ = 0.5, *P* = 0.5; repeated-measures 2-way ANOVA). **(G) Hunger** ratings remained stable from pre- to post-conditioning (t_(50)_ = 0.3, *P* = 0.8) as well as **(H) Thirst** ratings (t_(50)_ = 0.5, *P* = 0.6; paired *t* test). **(I) Novelty** ratings significantly decreased from pre to post-conditioning (F_(1,51)_ = 10.2, *P* = 0.002; post hoc CS^-^, *P* = 0.0001; post hoc CS^+^, *P* = 0.01) but similarly for both stimuli (F_(1, 51)_ = 0.17, *P* = 0.7; Interaction: F_(1, 51)_ = 1.1, *P* = 0.3). **(J) Intensity** ratings remained similar from pre- to post-conditioning (F_(1,51)_ = 0.6, *P* = 0.6), for both CS^-^ and CS^+^ flavors (F_(1,51)_ = 0.0003, *P* = 0.9; Interaction: F_(1,51)_ = 0.2, *P* = 0.7; repeated-measures 2-way ANOVA). Bar graphs represent the mean ± standard error of the mean (SEM). gLMS/gLHS, general labeled magnitude/hedonic scale; VAS, Visual Analogue Scale. *P ≤ 0.05; **P ≤ 0.01; ***P ≤ 0.001. The data supporting this figure is available in S1 Data.
(TIF)

S2 Fig Analyses excluding participants with BMI 25 kg/m^2^ or greater from healthy volunteer groups. **(A)** ΔCS+ preference intake, excluding healthy volunteers with BMI ≥ 25 kg/m^2^ from the FNC development group. Similarly to the original analyses in the full data set (Fig 1F), there was a significant post-conditioning increase in preference among participants with low preference at baseline (t_(23)_ = 2.7; *P* = 0.01; *n* = 24) but not in those with higher baseline preference (t_(19)_ = 0.05; *P* = 0.96; *n* = 20). **(B)** ΔCS+ preference pleasantness excluding healthy volunteers with BMI 25 kg/m^2^ or greater from the FNC development group. As found in the analyses with all participants (Fig 1F), ΔCS+ preference pleasantness did not change, irrespective of the baseline preference (low baseline pleasantness preference: t_(19)_ = 0.93; *P* = 0.4; *n* = 20; high baseline pleasantness preference: t_(23)_ = 1.85; *P* = 0.08; *n* = 24). **(C)** For participants in the clinical group, when excluding healthy volunteers with BMI ≥25 kg/m2, results were similar to those in the full data set (Fig 2A), with ΔCS+ preference increasing significantly after conditioning (t_(42)_ = 3.4, *P* = 0.0013; *n* = 43), and similarly across healthy (*n* = 14), obese (*n* = 9), and surgical (*n* = 20) participants (one-way ANOVA: F_(2, 41)_ = 1.55; *P* = 0.23, *n* = 43). **(D)** Similarly, for ΔCS+ preference pleasantness in the clinical group, when excluding healthy participants with BMI ≥25 kg/m2, and similarly to the original analyses (Fig 2B), post-conditioning changes were not significant (t_(42)_ = 0.7, *P* = 0.5; *n* = 43), and did not differ across groups (one-way ANOVA: F_(2, 41)_ = 0.11; *P* = 0.90, *n* = 43). **(E)** Striatal DD2lR availability for the clinical group when excluding healthy participants with BMI ≥25 kg/m^2^ revealed group effects similar to those found in the original analyses (Fig 3B), with significant overall effects in the one-way ANOVA (F_(2, 44)_ = 8.81, *P* = 0.0006, *n* = 46). Post hoc tests supported lower striatal binding potential for the obesity group relative to the surgical group (*P* = 0.0004), but differences relative to the healthy group did not reach significance (*P* = 0.1). The data supporting this figure is available in S1 Data.
(TIF)

S3 Fig Flow diagrams of the clinical study groups. **(A)** Flow diagram of recruitment of the healthy control group. Six volunteers did not perform SPECT due to failures in [^123^I]IBZM delivery or malfunction of the gamma camera. In 3 participants, data for FNC was not considered because there was an error in the preparation of solutions for the FNC protocol. **(B)** Flow diagram of the recruitment of the obesity and surgical groups. Across both groups, 4 participants were excluded from the analysis of FNC due to low at-home consumption of yogurt solutions for conditioning (2 in the Obese group and 2 in the Surgical group), and another participant was excluded due to an error in applying the FNC protocol. FNC, flavor-nutrient conditioning protocol; [^123^I]IBZM SPECT: [^123^I] iodobenzamide ([^123^I]IBZM) single-photon emission computed tomography (SPECT).
(TIF)

S4 Fig Intake and novelty ratings of CS- and CS+ flavors at baseline. As expected per experimental design, there were differences between the intake of flavors paired with maltodextrin (CS+) or with CMC (CS-) within the healthy, obese, and surgical groups (**A**) as shown by a mixed-model 2-way ANOVA significant for stimulus (i.e., CS- vs. CS+: F_(1, 50)_ = 71, *P* < 0.0001). However, there were no differences between groups, as shown by nonsignificant effects for Group (F_(2, 50)_ = 1.9, *P* = 0.16) nor the interaction between group and stimulus (F_(2, 50)_ = 0.6, *P* = 0.6). Post hoc tests showed significant differences between the intake of CS- and CS+ flavors within controls (mean difference = 31; 95% CI, 19.5 to 42.5, *P* < 0.0001), the obesity group (mean difference = 31; 95% CI, 12.2 to 49.8, *P* = 0.0005), and surgical group (mean difference = 24.2; 95% CI, 11.6 to 36.7, *P* < 0.0001). Regarding novelty ratings (**B**), a mixed-model 2-way ANOVA showed that ratings varied significantly according to stimulus (stimulus: F_(1, 100)_ = 7.1, *P* = 0.01), again with no significant effects for Group (F_(2, 100)_ = 2.7, *P* = 0.1) nor for interaction between group and stimulus (F_(2, 100)_ = 0.1, *P* = 0.9). However, post hoc tests showed that the stimulus effect was less robust than that for intake, with nonsignificant differences between the baseline novelty ratings of CS- and CS+ flavors within healthy, obese, and surgical groups (0.13< *P* <0.5). Bar graphs represent the mean ± standard error of the mean (SEM). VAS, Visual Analogue Scale. **P ≤ 0.01; ***P ≤ 0.001; ****P ≤ 0.0001. The data supporting this figure is available in S1 Data.
(TIF)

S5 Fig Complementary conditioning measures across the clinical study groups. Across conditioning for CS^-^ and CS^+^ flavors, ratings did not vary according to stimulus or group for **(A) Hunger** (Stimulus: F_(1, 50_ = 0.003, *P* = 0.96; Group: F_(2, 50)_ = 2.2, *P* = 0.1; Interaction: F_(2, 50)_ = 0.1, *P* = 0.7); **(B) Thirst** (Stimulus: F_(1, 50)_ = 0.01, *P* = 0.9; Group: F_(2, 50)_ = 1.5, *P* = 0.22; Interaction: F _(2, 50)_ = 0.96, *P* = 0.4) and **(C) Novelty** (Stimulus: F_(1, 50)_ = 1.6, *P* = 0.2; Group: F_(2, 50)_ = 1.8, *P* = 0.17; Interaction: F_(2, 50)_ = 0.5, *P* = 0.6). (**D**) **Intensity** ratings were different in CS^-^ vs. CS^+^ (F_(1, 50)_ = 5.2, *P* = 0.03), with a no significant effects for Group (F_(2, 50)_ = 0.1, *P* = 0.9) nor for interaction (F_(2, 50)_ = 0.9, *P* = 0.4). (**E**) **Pleasantness** ratings, however, did not differ according to stimulus (F_1, 50)_ = 0.5, *P* = 0.5) nor according to group (F_(1, 50)_ = 0.7, *P* = 0.5; Interaction: F_(2, 50)_ = 2.5, *P* = 0.1). **(F) Intake** was similar across conditioning for CS^-^ and CS^+^ flavors (F_(1, 50)_ = 0.5, *P* = 0.5) and despite findings of a significant group effect (F_(1, 50)_ = 7.5, *P* = 0.001), interaction between factors was not significant (F_(2, 50)_ = 1.0, *P* = 0.4; mixed-model 2-way ANOVA). From pre- to post-conditioning days, ratings remained stable and did not vary according to group for **(G) Hunger** (Time: F_(1, 47)_ = 0.2, *P* = 0.6; Group: F_(2, 50)_ = 1.98, *P* = 0.2; Interaction: F_(2, 47)_ = 0.3, *P* = 0.7) and **(H) Thirst** (Time: F _(1, 47)_ = 0.05, *P* = 0.8; Group: F_(2, 50)_ = 2.5, *P* = 0.1; Interaction: F_(2, 47)_ = 0.8; *P* = 0.5). **(I) Novelty** ratings changed from pre to post-conditioning (F_(1, 50)_ = 12.9, *P* = 0.001), with a significant effect for group (F_(2, 50)_ = 3.4, *P* = 0.04) and a nonsignificant interaction between factors (F_(2, 50)_ = 0.7; *P* = 0.5). Post hoc tests showed significant decreases for the surgical group (*P* = 0.05), while in the remaining groups, results did not reach significance (Healthy, *P* = 0.4; Obese, *P* = 0.1). **(J) Intensity** ratings remained similar from pre- to post-conditioning (F_(1, 50)_ = 0.7, *P* = 0.4), with no effects for group (F_(2, 50)_ = 0.66, *P* = 0.52) nor interaction (F_(2, 50)_ = 1.5; *P* = 0.2; mixed-model 2-way ANOVA). Bar graphs represent the mean ± standard error of the mean (SEM). gLMS/ gLHS, general labeled magnitude/hedonic scale; VAS, Visual Analogue Scale. *P ≤ 0.05; ***P ≤ 0.001; ****P ≤ 0.0001. The data supporting this figure is available in S1 Data.
(TIF)

S1 Table Demographic characteristics of healthy subjects. (TIF)

S2 Table Gustatory and psychometric measures of feeding behavior in healthy subjects. (TIF)

S3 Table Associations between striatal dopamine DD2lR availability, conditioning strength, and feeding behavior. (TIF)

S4 Table Details of the primary statistical models performed. (TIF)

S5 Table Effect sizes of the primary statistical models performed. (TIF)

S1 Data Individual quantitative observations that underlie the data summarized in main and supplementary figures and results. (XLSX)

## Abbreviations

3-AFC: 3-alternative forced-choice
3D-OSEM: 3D ordered subsets expectation maximization
BMI: body mass index
BP: binding potential
CMC: carboxymethylcellulose
DD2lR: dopamine D2-like receptor
DE: dextrose equivalent
DEBQ: Dutch Eating Behaviour Questionnaire
FARS: Food Action Rating Scale
FNC: flavornutrient conditioning
gLMS/gLHS: general labeled magnitude/hedonic scales
PET: positron emission tomography
PFS: Power of Food Scale
ROI: region of interest
SEM: standard error of the mean
SPECT: single-photon emission computed tomography
T2DM: Type 2 diabetes mellitus
VAS: Visual Analogue Scales
VTA: ventral tegmental area
YFAS: Yale Food Addiction Scale
