# Supplementary material for: Postingestive reward acts through behavioral reinforcement and is conserved in obesity and after bariatric surgery
Source: PLoS Biol. 2024 Dec 17;22(12):e3002936. doi: 10.1371/journal.pbio.3002936 (PMC11651594; doi:10.1371/journal.pbio.3002936)
Supplement: S1 STROBE Checklist — (DOC) [file pbio.3002936.s001.doc]

STROBE Statement—Checklist of items that should be included in reports of ***cross-sectional studies***

|  | Item No | Recommendation |  |
| --- | --- | --- | --- |
| **Title and abstract** | 1 | (*a*) Indicate the study’s design with a commonly used term in the title or the abstract | Line 70 |
|  |  | (*b*) Provide in the abstract an informative and balanced summary of what was done and what was found | Line 70-83 |
| Introduction | | |  |
| Background/rationale | 2 | Explain the scientific background and rationale for the investigation being reported | Line 88-120 |
| Objectives | 3 | State specific objectives, including any prespecified hypotheses | Line 122-131 |
| Methods | | |  |
| Study design | 4 | Present key elements of study design early in the paper | Line 135 |
| Setting | 5 | Describe the setting, locations, and relevant dates, including periods of recruitment, exposure, follow-up, and data collection | Line 462-494 |
| Participants | 6 | (*a*) Give the eligibility criteria, and the sources and methods of selection of participants | Line 462-494 |
| Variables | 7 | Clearly define all outcomes, exposures, predictors, potential confounders, and effect modifiers. Give diagnostic criteria, if applicable | Line 460-486 |
| Data sources/ measurement | 8* | For each variable of interest, give sources of data and details of methods of assessment (measurement). Describe comparability of assessment methods if there is more than one group | Line 496-617 |
| Bias | 9 | Describe any efforts to address potential sources of bias | Line 461-485 |
| Study size | 10 | Explain how the study size was arrived at | Supplementary Figure 3. |
| Quantitative variables | 11 | Explain how quantitative variables were handled in the analyses. If applicable, describe which groupings were chosen and why | Line 619-659  Supplementary Table 4 |
| Statistical methods | 12 | (*a*) Describe all statistical methods, including those used to control for confounding | Line 619-659  Supplementary Table 4 |
|  |  | (*b*) Describe any methods used to examine subgroups and interactions | Line 619-659  Supplementary Table 4 |
|  |  | (*c*) Explain how missing data were addressed | Line 621 - 624 |
|  |  | (*d*) If applicable, describe analytical methods taking account of sampling strategy | Not applicable |
|  |  | (*e*) Describe any sensitivity analyses | Supplementary Tables 4 |
| Results | | |  |
| Participants | 13* | (a) Report numbers of individuals at each stage of study—eg numbers potentially eligible, examined for eligibility, confirmed eligible, included in the study, completing follow-up, and analysed | Supplementary Figure 3. |
|  |  | (b) Give reasons for non-participation at each stage | Supplementary Figure 3. |
|  |  | (c) Consider use of a flow diagram | Supplementary Figure 3. |
| Descriptive data | 14* | (a) Give characteristics of study participants (eg demographic, clinical, social) and information on exposures and potential confounders | Table 1 and Supplementary Table 1. |
|  |  | (b) Indicate number of participants with missing data for each variable of interest | Figure 1-3. |
| Outcome data | 15* | Report numbers of outcome events or summary measures | Supplementary Table 5 |
| Main results | 16 | (*a*) Give unadjusted estimates and, if applicable, confounder-adjusted estimates and their precision (eg, 95% confidence interval). Make clear which confounders were adjusted for and why they were included | Supplementary Tables 4-5 |
|  |  | (*b*) Report category boundaries when continuous variables were categorized | Not applicable |
|  |  | (*c*) If relevant, consider translating estimates of relative risk into absolute risk for a meaningful time period | Not applicable |
| Other analyses | 17 | Report other analyses done—eg analyses of subgroups and interactions, and sensitivity analyses | Supplementary Tables 4 |
| Discussion | | |  |
| Key results | 18 | Summarise key results with reference to study objectives | Line 301-311 |
| Limitations | 19 | Discuss limitations of the study, taking into account sources of potential bias or imprecision. Discuss both direction and magnitude of any potential bias | Line 419-446 |
| Interpretation | 20 | Give a cautious overall interpretation of results considering objectives, limitations, multiplicity of analyses, results from similar studies, and other relevant evidence | Line 313-419 |
| Generalisability | 21 | Discuss the generalisability (external validity) of the study results | Line 433-436 |
| Other information | | | Line 443-446 |
| Funding | 22 | Give the source of funding and the role of the funders for the present study and, if applicable, for the original study on which the present article is based | Line 668-680 |

*Give information separately for exposed and unexposed groups.

**Note:** An Explanation and Elaboration article discusses each checklist item and gives methodological background and published examples of transparent reporting. The STROBE checklist is best used in conjunction with this article (freely available on the Web sites of PLoS Medicine at http://www.plosmedicine.org/, Annals of Internal Medicine at http://www.annals.org/, and Epidemiology at http://www.epidem.com/). Information on the STROBE Initiative is available at www.strobe-statement.org.
