# Supplementary figures and images for: Postingestive reward acts through behavioral reinforcement and is conserved in obesity and after bariatric surgery
Source: PLoS Biol. 2024 Dec 17;22(12):e3002936. doi: 10.1371/journal.pbio.3002936 (PMC11651594; doi:10.1371/journal.pbio.3002936)

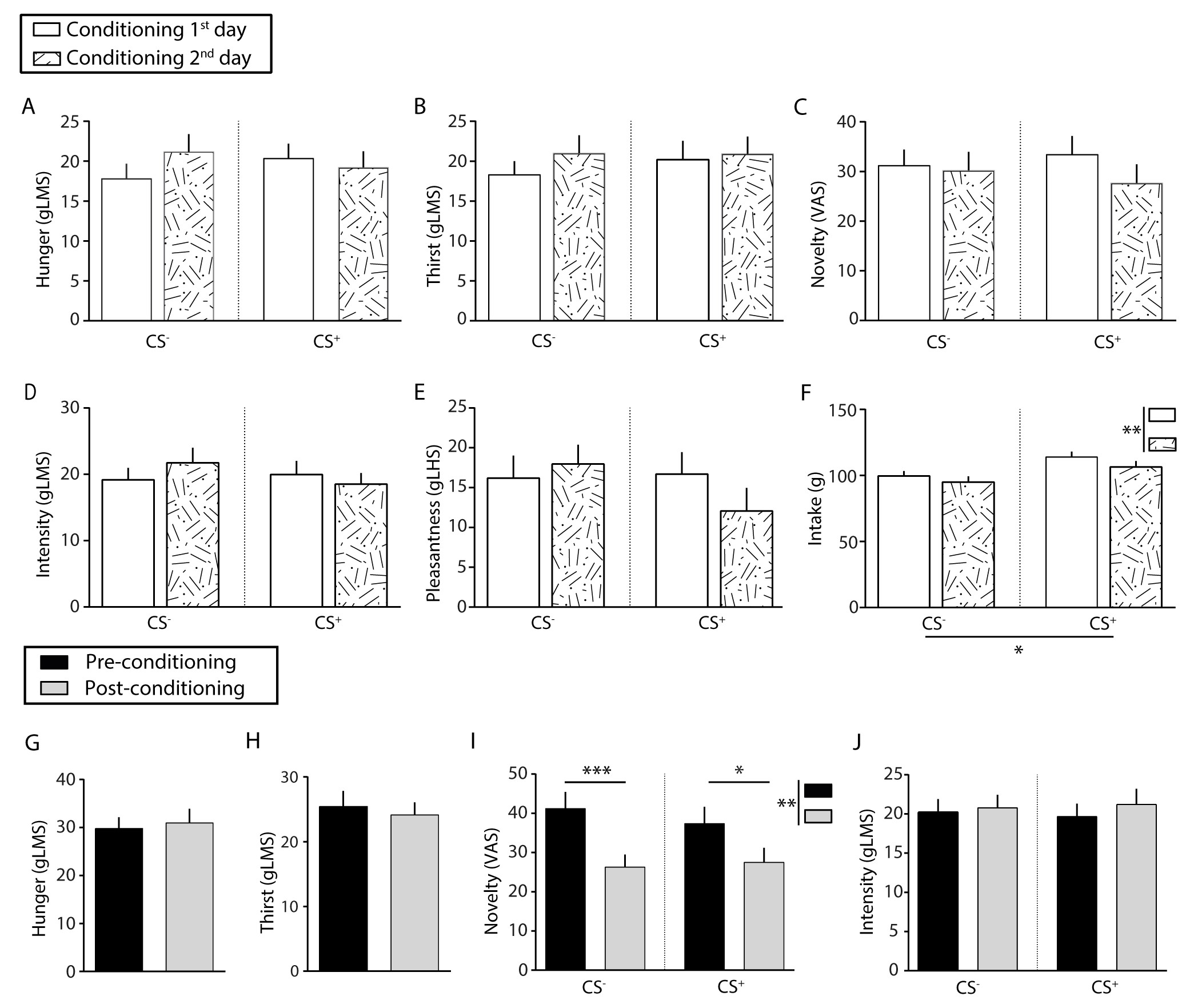

Supplement: S1 Fig — Across conditioning for CS- and CS+ flavors, ratings did not vary according to stimulus or conditioning day for (A) Hunger (Stimulus: F(1,102) = 0.04, P = 0.8; Day: F(1, 102) = 3.0, P = 0.08; Interaction: F(1,102) = 9.9, P = 0.9), (B) Thirst (Stimulus: F(1,102) = 0.1, p = 0.8; Day: F(1, 102) = 3.2, P = 0.07; Interaction: F(1,102) = 0.08, P = 0.8), (C) Novelty (Stimulus: F(1,102) = 0.002, P = 0.9; Day: F(1, 102) = 2.7, P = 0.1; Interaction: F(1,102) = 1.3, P = 0.3), (D) Intensity (Stimulus: F(1,99) = 0.3, P = 0.6; Day: F(1, 99) = 0.09, P = 0.8; Interaction: F(1,99) = 1.2, P = 0.3), and (E) Pleasantness (Stimulus: F(1,98) = 0.9, P = 0.3; Day: F(1,98)) = 0.4, P = 0.5; Interaction: F(1,98) = 1.9, P = 0.2). (F) Intake volumes were higher for CS+ than CS- (F(1,102) = 5.5, P = 0.02), and decreased across conditioning days (F(1,102) = 8.1, P = 0.005; Interaction: F(1,102) = 0.5, P = 0.5; repeated-measures 2-way ANOVA). (G) Hunger ratings remained stable from pre- to post-conditioning (t(50) = 0.3, P = 0.8) as well as (H) Thirst ratings (t(50) = 0.5, P = 0.6; paired t test). (I) Novelty ratings significantly decreased from pre to post-conditioning (F(1,51) = 10.2, P = 0.002; post hoc CS-, P = 0.0001; post hoc CS+, P = 0.01) but similarly for both stimuli (F(1, 51) = 0.17, P = 0.7; Interaction: F(1, 51) = 1.1, P = 0.3). (J) Intensity ratings remained similar from pre- to post-conditioning (F(1,51) = 0.6, P = 0.6), for both CS- and CS+ flavors (F(1,51) = 0.0003, P = 0.9; Interaction: F(1,51) = 0.2, P = 0.7; repeated-measures 2-way ANOVA). Bar graphs represent the mean ± standard error of the mean (SEM). gLMS/gLHS, general labeled magnitude/hedonic scale; VAS, Visual Analogue Scale. *P ≤ 0.05; **P ≤ 0.01; ***P ≤ 0.001. The data supporting this figure is available in S1 Data. (TIF) [file pbio.3002936.s002.tif]

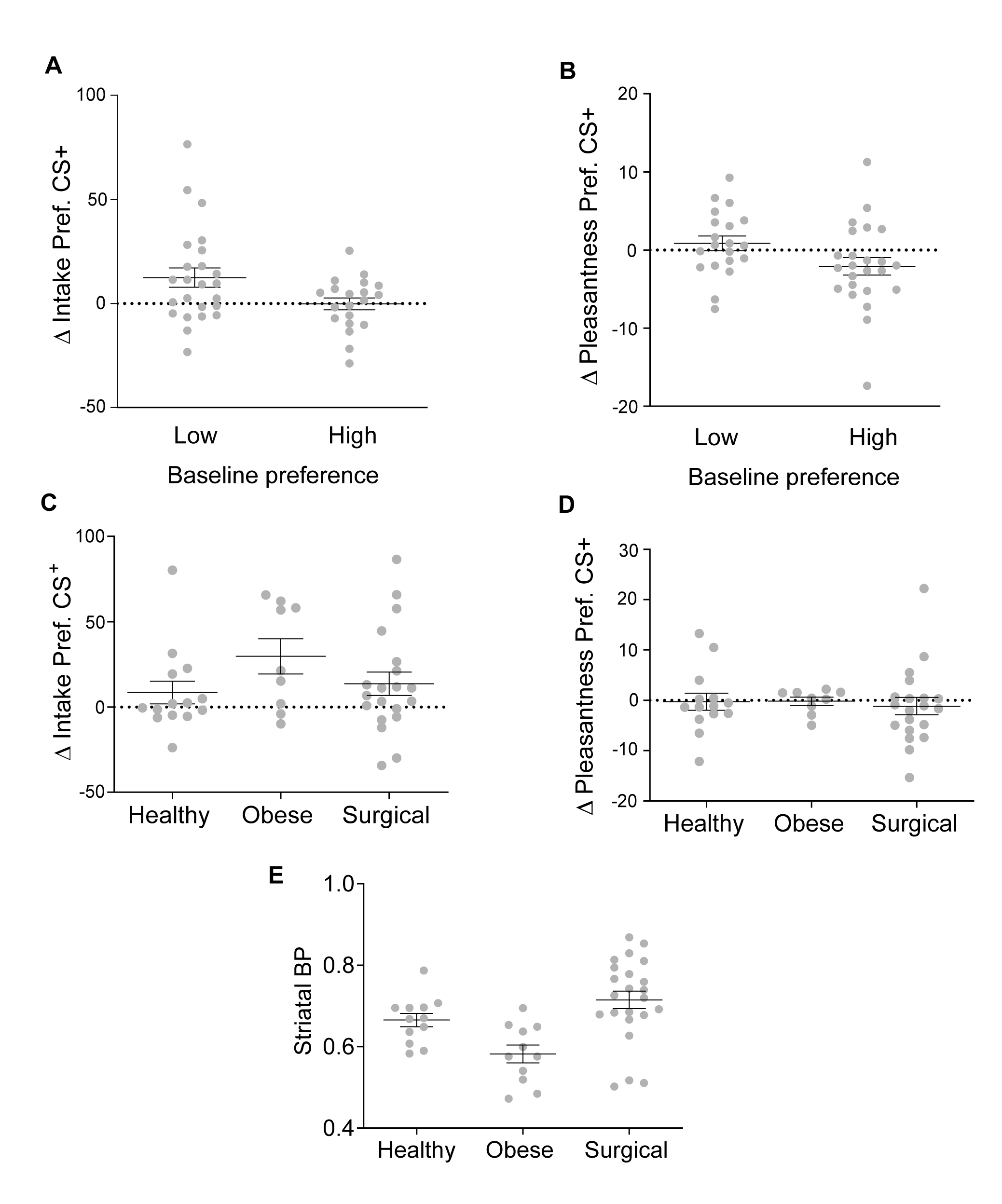

Supplement: S2 Fig — (A) ΔCS+ preference intake, excluding healthy volunteers with BMI ≥ 25 kg/m2 from the FNC development group. Similarly to the original analyses in the full data set (Fig 1F), there was a significant post-conditioning increase in preference among participants with low preference at baseline (t(23) = 2.7; P = 0.01; n = 24) but not in those with higher baseline preference (t(19) = 0.05; P = 0.96; n = 20). (B) ΔCS+ preference pleasantness excluding healthy volunteers with BMI 25 kg/m2 or greater from the FNC development group. As found in the analyses with all participants (Fig 1F), ΔCS+ preference pleasantness did not change, irrespective of the baseline preference (low baseline pleasantness preference: t(19) = 0.93; P = 0.4; n = 20; high baseline pleasantness preference: t(23) = 1.85; P = 0.08; n = 24). (C) For participants in the clinical group, when excluding healthy volunteers with BMI ≥25 kg/m2, results were similar to those in the full data set (Fig 2A), with ΔCS+ preference increasing significantly after conditioning (t(42) = 3.4, P = 0.0013; n = 43), and similarly across healthy (n = 14), obese (n = 9), and surgical (n = 20) participants (one-way ANOVA: F(2, 41) = 1.55; P = 0.23, n = 43). (D) Similarly, for ΔCS+ preference pleasantness in the clinical group, when excluding healthy participants with BMI ≥25 kg/m2, and similarly to the original analyses (Fig 2B), post-conditioning changes were not significant (t(42) = 0.7, P = 0.5; n = 43), and did not differ across groups (one-way ANOVA: F(2, 41) = 0.11; P = 0.90, n = 43). (E) Striatal DD2lR availability for the clinical group when excluding healthy participants with BMI ≥25 kg/m2 revealed group effects similar to those found in the original analyses (Fig 3B), with significant overall effects in the one-way ANOVA (F(2, 44) = 8.81, P = 0.0006, n = 46). Post hoc tests supported lower striatal binding potential for the obesity group relative to the surgical group (P = 0.0004), but differences relative to the health [file pbio.3002936.s003.tif]

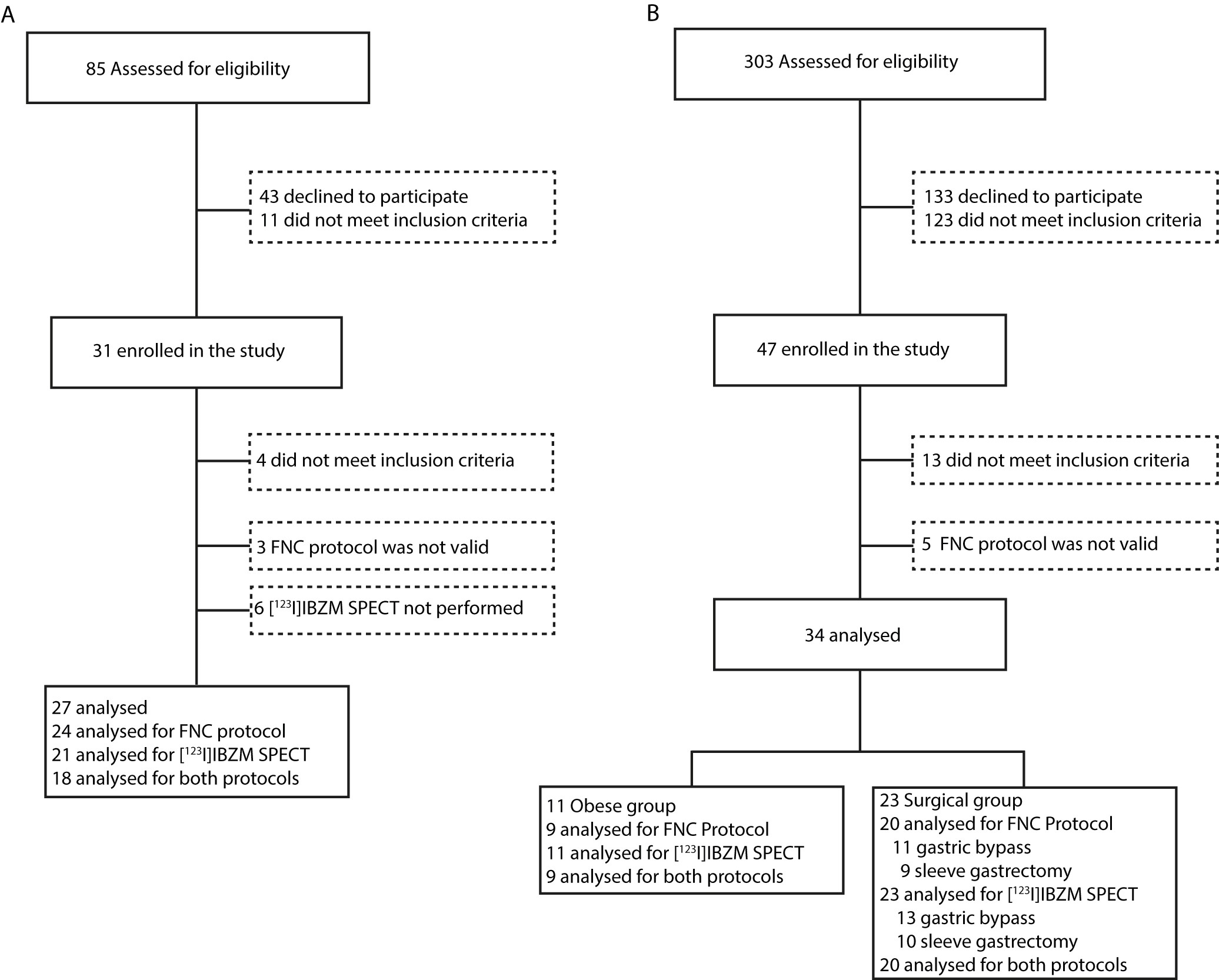

Supplement: S3 Fig — (A) Flow diagram of recruitment of the healthy control group. Six volunteers did not perform SPECT due to failures in [123I]IBZM delivery or malfunction of the gamma camera. In 3 participants, data for FNC was not considered because there was an error in the preparation of solutions for the FNC protocol. (B) Flow diagram of the recruitment of the obesity and surgical groups. Across both groups, 4 participants were excluded from the analysis of FNC due to low at-home consumption of yogurt solutions for conditioning (2 in the Obese group and 2 in the Surgical group), and another participant was excluded due to an error in applying the FNC protocol. FNC, flavor-nutrient conditioning protocol; [123I]IBZM SPECT: [123I] iodobenzamide ([123I]IBZM) single-photon emission computed tomography (SPECT). (TIF) [file pbio.3002936.s004.tif]

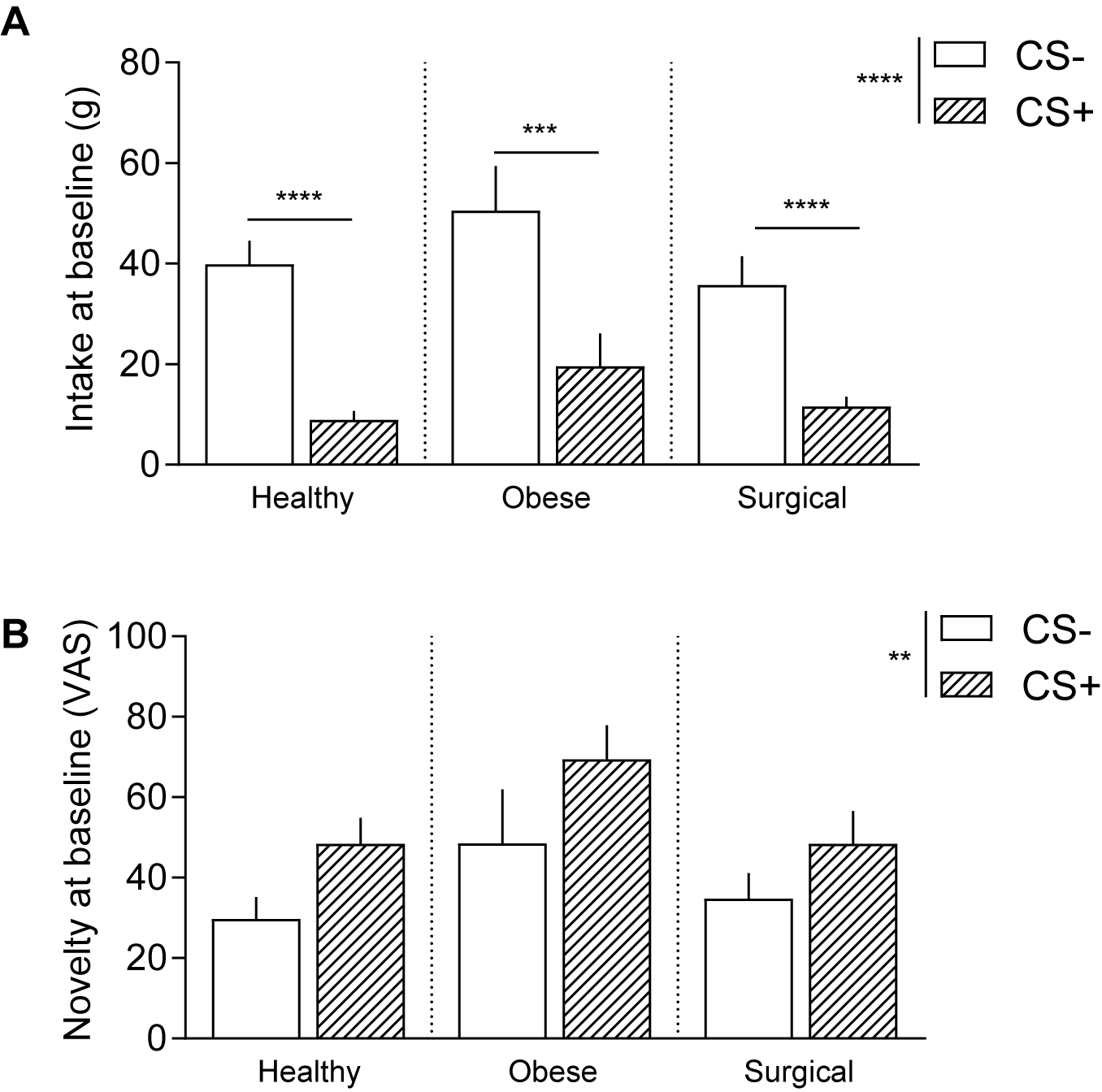

Supplement: S4 Fig — As expected per experimental design, there were differences between the intake of flavors paired with maltodextrin (CS+) or with CMC (CS-) within the healthy, obese, and surgical groups (A) as shown by a mixed-model 2-way ANOVA significant for stimulus (i.e., CS- vs. CS+: F(1, 50) = 71, P < 0.0001). However, there were no differences between groups, as shown by nonsignificant effects for Group (F(2, 50) = 1.9, P = 0.16) nor the interaction between group and stimulus (F(2, 50) = 0.6, P = 0.6). Post hoc tests showed significant differences between the intake of CS- and CS+ flavors within controls (mean difference = 31; 95% CI, 19.5 to 42.5, P < 0.0001), the obesity group (mean difference = 31; 95% CI, 12.2 to 49.8, P = 0.0005), and surgical group (mean difference = 24.2; 95% CI, 11.6 to 36.7, P < 0.0001). Regarding novelty ratings (B), a mixed-model 2-way ANOVA showed that ratings varied significantly according to stimulus (stimulus: F(1, 100) = 7.1, P = 0.01), again with no significant effects for Group (F(2, 100) = 2.7, P = 0.1) nor for interaction between group and stimulus (F(2, 100) = 0.1, P = 0.9). However, post hoc tests showed that the stimulus effect was less robust than that for intake, with nonsignificant differences between the baseline novelty ratings of CS- and CS+ flavors within healthy, obese, and surgical groups (0.13< P <0.5). Bar graphs represent the mean ± standard error of the mean (SEM). VAS, Visual Analogue Scale. **P ≤ 0.01; ***P ≤ 0.001; ****P ≤ 0.0001. The data supporting this figure is available in S1 Data. (TIF) [file pbio.3002936.s005.tif]

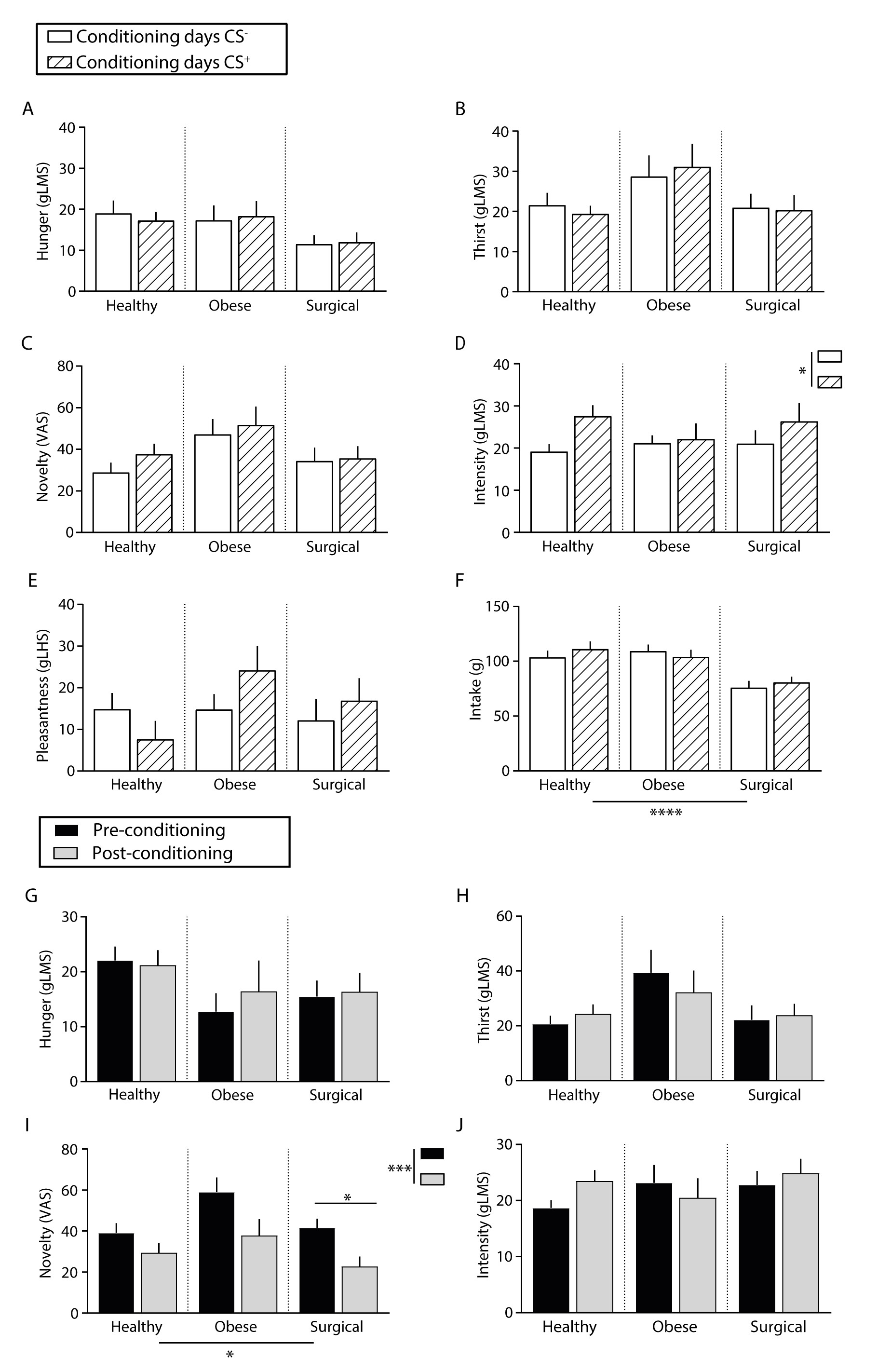

Supplement: S5 Fig — Across conditioning for CS- and CS+ flavors, ratings did not vary according to stimulus or group for (A) Hunger (Stimulus: F(1, 50 = 0.003, P = 0.96; Group: F(2, 50) = 2.2, P = 0.1; Interaction: F(2, 50) = 0.1, P = 0.7); (B) Thirst (Stimulus: F(1, 50) = 0.01, P = 0.9; Group: F(2, 50) = 1.5, P = 0.22; Interaction: F (2, 50) = 0.96, P = 0.4) and (C) Novelty (Stimulus: F(1, 50) = 1.6, P = 0.2; Group: F(2, 50) = 1.8, P = 0.17; Interaction: F(2, 50) = 0.5, P = 0.6). (D) Intensity ratings were different in CS- vs. CS+ (F(1, 50) = 5.2, P = 0.03), with a no significant effects for Group (F(2, 50) = 0.1, P = 0.9) nor for interaction (F(2, 50) = 0.9, P = 0.4). (E) Pleasantness ratings, however, did not differ according to stimulus (F1, 50) = 0.5, P = 0.5) nor according to group (F(1, 50) = 0.7, P = 0.5; Interaction: F(2, 50) = 2.5, P = 0.1). (F) Intake was similar across conditioning for CS- and CS+ flavors (F(1, 50) = 0.5, P = 0.5) and despite findings of a significant group effect (F(1, 50) = 7.5, P = 0.001), interaction between factors was not significant (F(2, 50) = 1.0, P = 0.4; mixed-model 2-way ANOVA). From pre- to post-conditioning days, ratings remained stable and did not vary according to group for (G) Hunger (Time: F(1, 47) = 0.2, P = 0.6; Group: F(2, 50) = 1.98, P = 0.2; Interaction: F(2, 47) = 0.3, P = 0.7) and (H) Thirst (Time: F (1, 47) = 0.05, P = 0.8; Group: F(2, 50) = 2.5, P = 0.1; Interaction: F(2, 47) = 0.8; P = 0.5). (I) Novelty ratings changed from pre to post-conditioning (F(1, 50) = 12.9, P = 0.001), with a significant effect for group (F(2, 50) = 3.4, P = 0.04) and a nonsignificant interaction between factors (F(2, 50) = 0.7; P = 0.5). Post hoc tests showed significant decreases for the surgical group (P = 0.05), while in the remaining groups, results did not reach significance (Healthy, P = 0.4; Obese, P = 0.1). (J) Intensity ratings remained similar from pre- to post-conditioning (F(1, 50) = 0.7, P = 0.4), with no effects for group (F(2, 50) = 0.66, [file pbio.3002936.s006.tif]

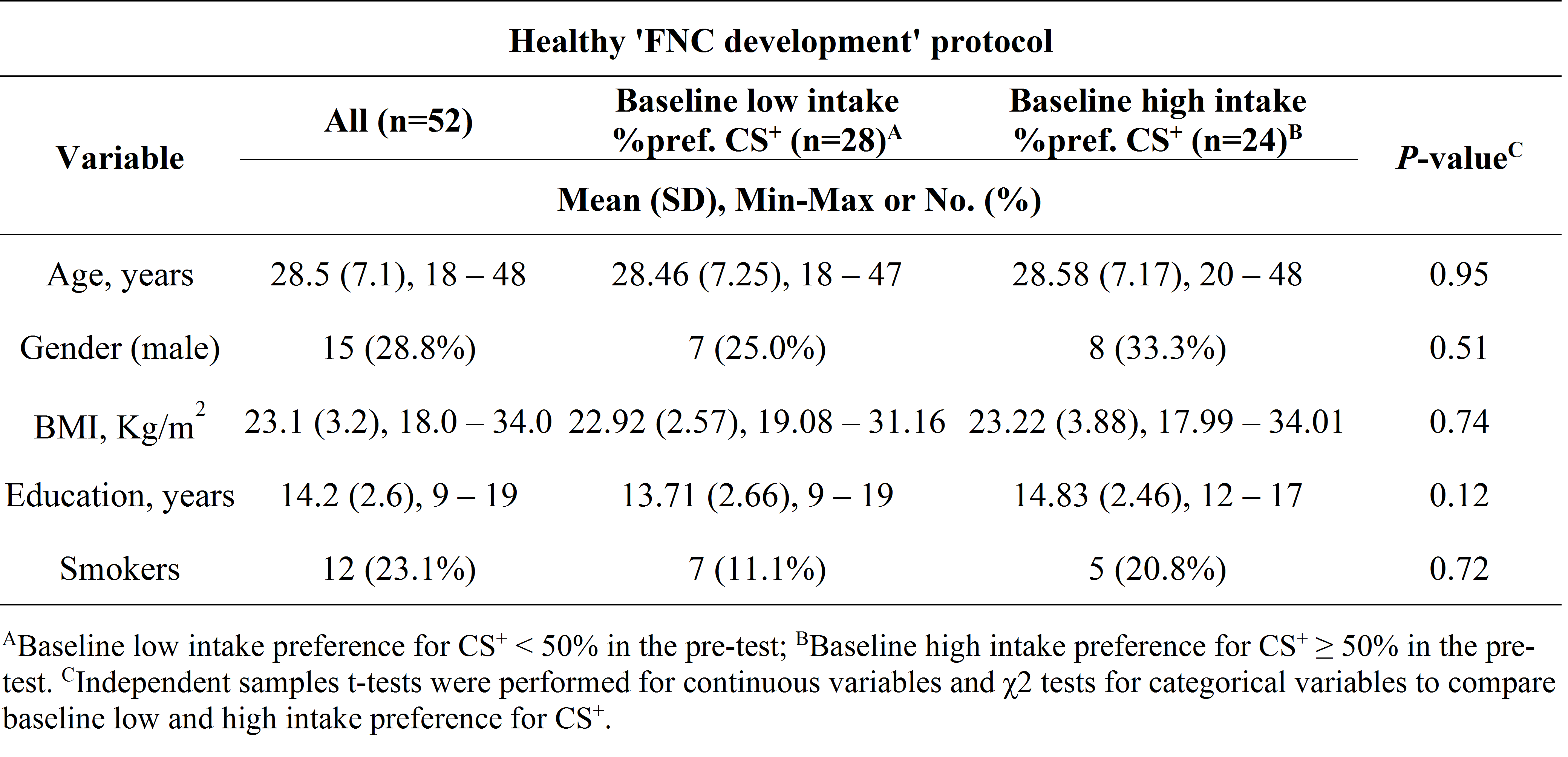

Supplement: S1 Table — (TIF) [file pbio.3002936.s007.tif]

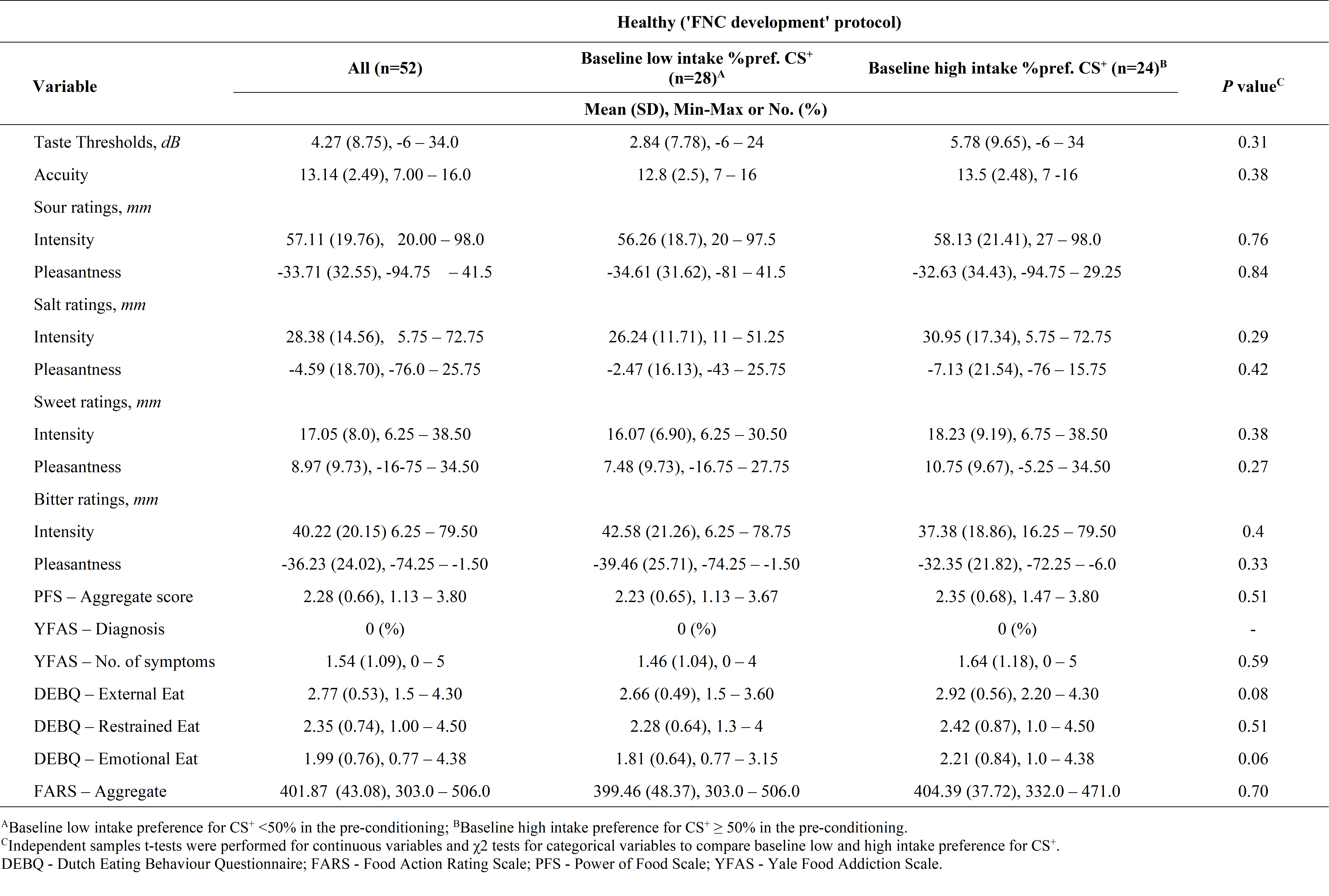

Supplement: S2 Table — (TIF) [file pbio.3002936.s008.tif]

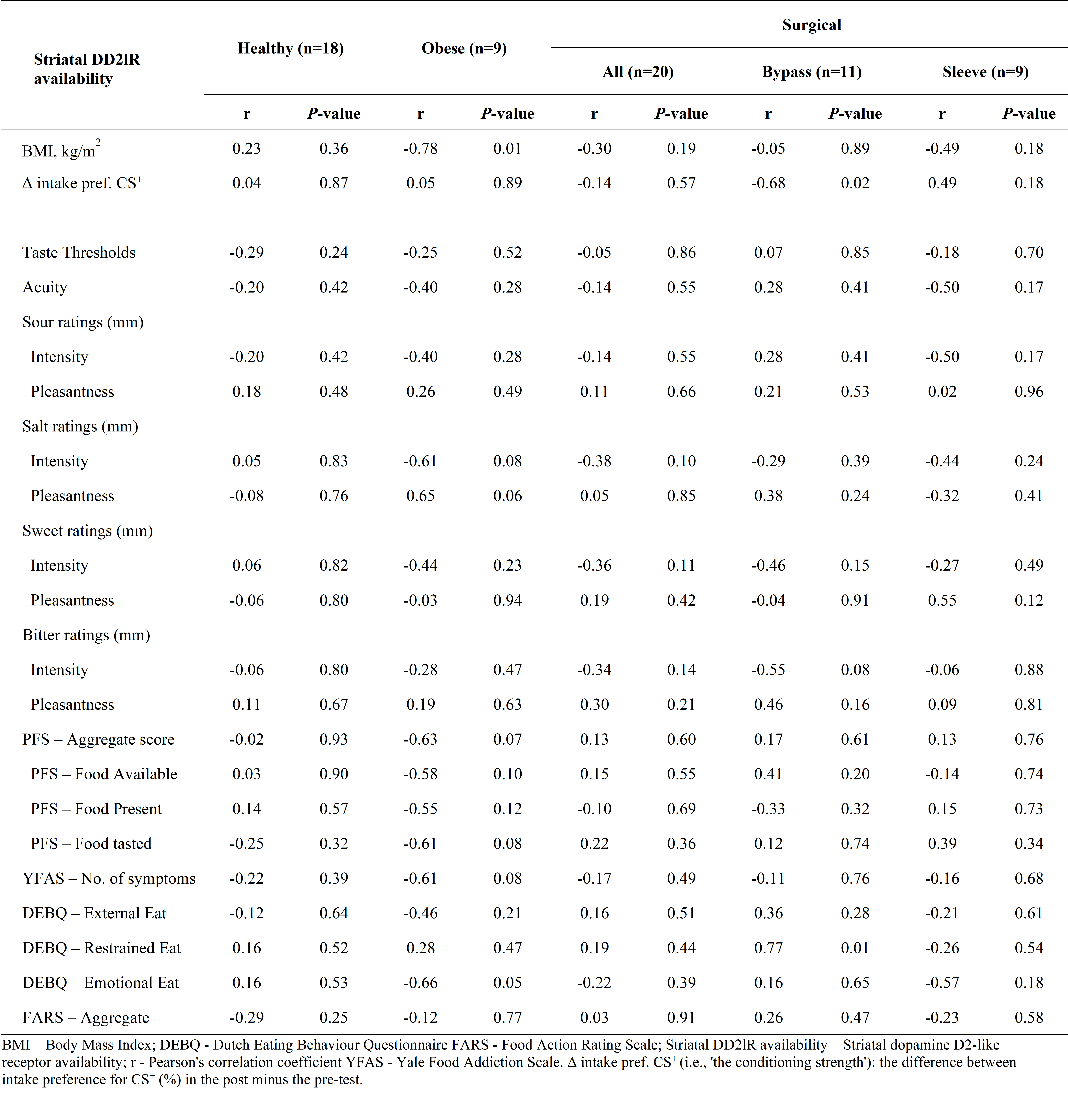

Supplement: S3 Table — (TIF) [file pbio.3002936.s009.tif]

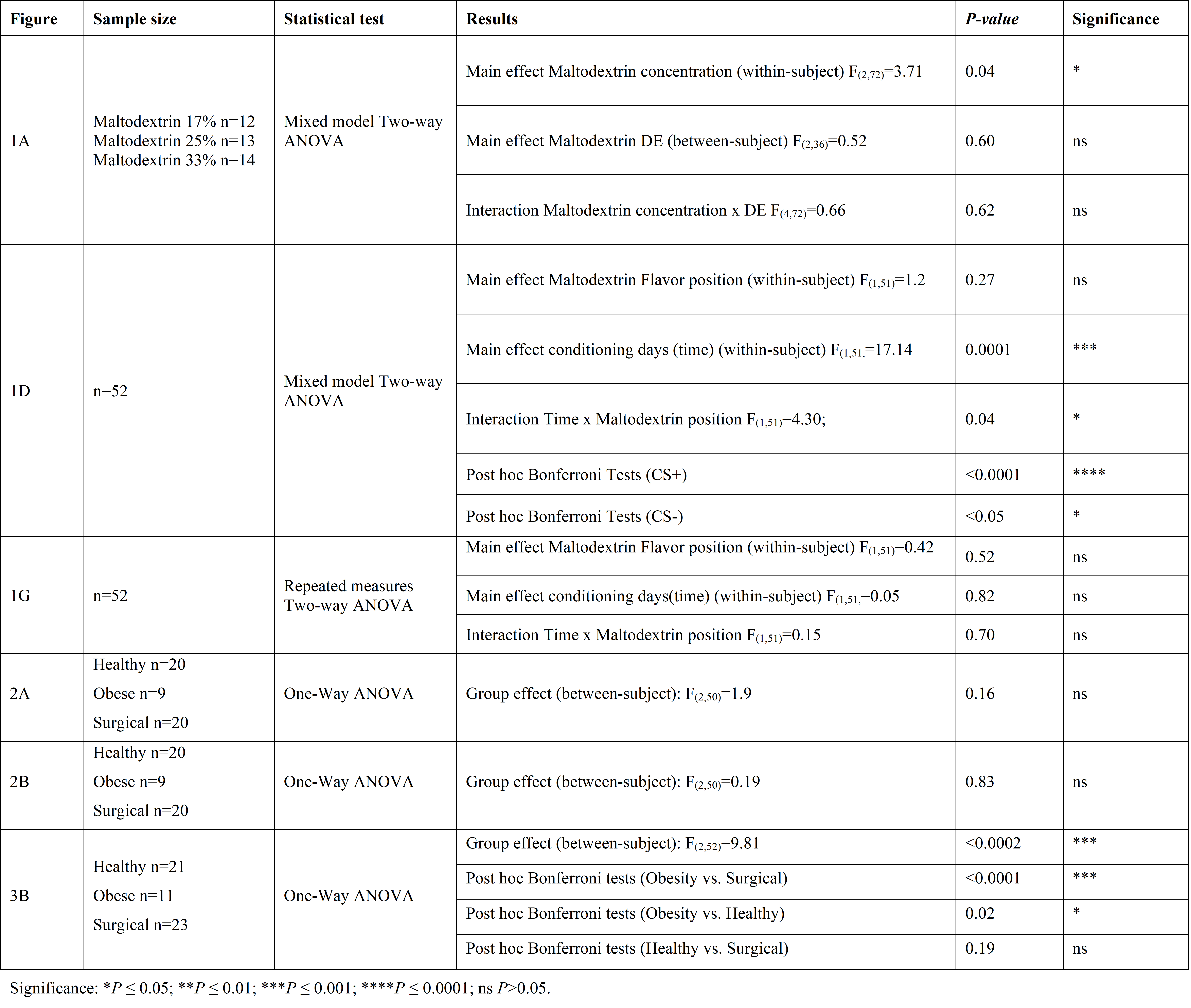

Supplement: S4 Table — (TIF) [file pbio.3002936.s010.tif]

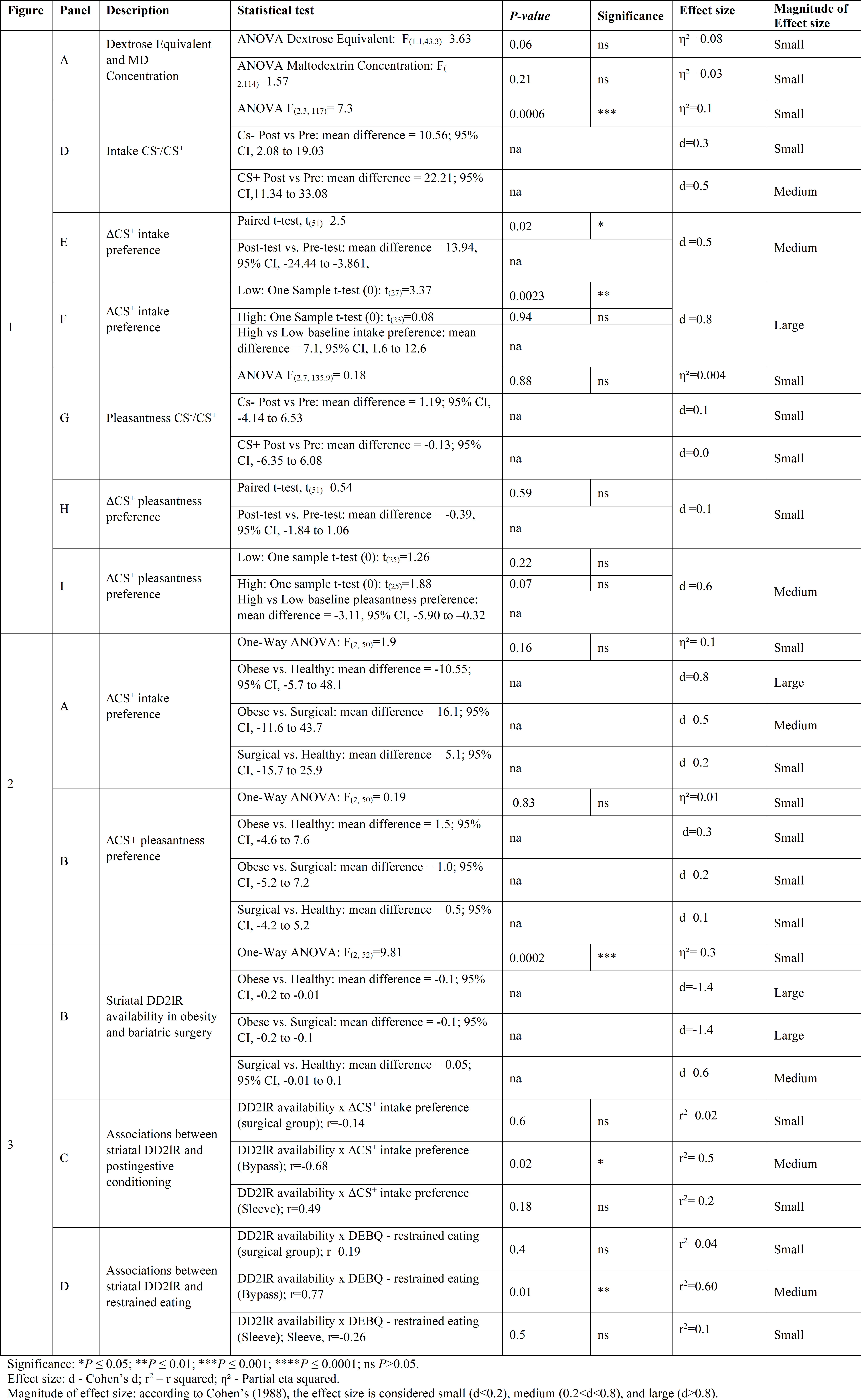

Supplement: S5 Table — (TIF) [file pbio.3002936.s011.tif]
